# The γ-Adducin 1–357 fragment promotes tau pathology

**DOI:** 10.3389/fnagi.2023.1241750

**Published:** 2023-09-13

**Authors:** Honglu Yu, Min Xiong, Congcong Liu, Danhao Xia, Lanxia Meng, Zhentao Zhang

**Affiliations:** ^1^Department of Neurology, Renmin Hospital of Wuhan University, Wuhan, China; ^2^TaiKang Center for Life and Medical Sciences, Wuhan University, Wuhan, China

**Keywords:** Alzheimer’s disease, γ-adducin, tau phosphorylation, glycogen synthase kinase-3β, 4-Benzyl-2-methyl-1,2,4-thiadiazolidine-3,5-dione

## Abstract

**Background:**

Tau phosphorylation is a pathological hallmark of Alzheimer’s disease (AD). Previously, we reported that the γ-adducin 1–357 fragment is present in the brains of AD patients. However, it remains unknown how γ-adducin regulates tau phosphorylation.

**Objective:**

The aim of this project is to investigate the effects of the γ-adducin 1–357 fragment on tau phosphorylation and the kinases involved in this process.

**Methods:**

Full-length γ-adducin or the γ-adducin 1–357 fragment was expressed in HEK293 cells, SH-SY5Y cells, and primary neurons. The phosphorylation of tau Ser396 was determined using Western blot and immunofluorescence. Tau P301S transgenic mice were injected with adeno-associated virus encoding full-length γ-adducin or γ-adducin 1–357 fragment to determine the phosphorylation of tau.

**Results:**

The γ-adducin 1–357 fragment enhances tau phosphorylation at Ser396. Additionally, the expression of the γ-adducin 1–357 fragment leads to the activation of glycogen synthase kinase-3β (GSK-3β). This effect was mitigated by the GSK-3β inhibitor 4-Benzyl-2-methyl-1,2,4-thiadiazolidine-3,5-dione (TDZD-8).

**Conclusion:**

The γ-adducin 1–357 fragment enhances tau phosphorylation by activating GSK3β. These results support that the fragmentation of γ-adducin may play a pivotal role in tau pathology.

## Introduction

Alzheimer’s disease (AD) is an age-related neurodegenerative disease characterized by memory loss and cognitive impairment (Scheltens et al., 2021). One of its key features is the presence of neurofibrillary tangles (NFTs), abnormal accumulations of tau protein with excessive phosphorylation (Metaxas and Kempf, 2016). Tau, a microtubule-associated protein (MAP), plays a central role in orchestrating the assembly, stabilization, and modulation of microtubules, which are vital for neuronal and cognitive functions (Wang and Mandelkow, 2016). Under pathological conditions, tau undergoes complex changes such as truncation and phosphorylation, forming harmful oligomers and fibrils (Li et al., 2022). Aspartic endopeptidase (AEP) has been reported to cleave tau and promote its aggregation (Zhang et al., 2014). Phosphorylation is one of the most prevalent post-translational modifications of tau in AD (Johnson and Stoothoff, 2004). Phosphorylation of tau facilitates the creation of pathological tau oligomers, which trigger neurotoxicity (Shafiei et al., 2017; Carroll et al., 2021). Understanding the molecular mechanisms underlying tau pathology will facilitate the development of novel therapeutic strategies.

Adducin is a widely expressed heterodimeric cytoskeletal protein composed of α-adducin (ADD1), β-adducin (ADD2), and γ-adducin (ADD3) subunits (Joshi et al., 1991). It plays a vital role in multiple biological processes, such as cytoskeletal organization, membrane trafficking, signal transduction, cell-to-cell contact formation, and cell migration (Matsuoka et al., 2000; Hiermaier et al., 2021). Specifically, γ-adducin is present at spectrin-actin junctions in the dendritic spines of hippocampal neurons, where it regulates synaptic plasticity, neurite growth, and degeneration (Matsuoka et al., 1998; Xu et al., 2013).

Previously, we reported that the γ-adducin 1–357 fragment induces neurite damage and synaptic dysfunction, promoting cognitive impairments in AD (Xiong et al., 2021). However, how the γ-adducin 1–357 fragment regulates tau phosphorylation remains unclear. Glycogen synthase kinase-3β (GSK-3β) is one of the primary kinases for the tau protein and is capable of phosphorylating tau at multiple serine and threonine residues (Sayas and Ávila, 2021). Here, we established cellular and animal models and observed that the γ-adducin 1–357 fragment significantly enhances tau phosphorylation at Ser396 by activating GSK-3β.

## Materials and methods

### Animals

Tau P301S transgenic mice on a C57BL/6 J background were obtained from Jackson Laboratory and housed at the Animal Experiment Center of Renmin Hospital, Wuhan University. The mice were maintained under specific pathogen-free (SPF) conditions with a 14-h light and 10-h dark cycle and had *ad libitum* access to food and water. All experimental procedures were approved by the Institutional Animal Care and Use Committee (IACUC) of Renmin Hospital, Wuhan University (IACUC issue number: WDRM animal [welfare] 202,200,084).

### AAV packaging and stereotaxic injection

Adeno-associated virus (AAV) particles encoding full-length γ-adducin and the γ-adducin 1–357 fragment under the control of the human synapsin I promoter were prepared by BrainVTA (Wuhan) Co., Ltd. Bilateral intracerebral injection of AAVs was performed stereotactically in two-month-old Tau P301S mice. The mice were first prepared by removing the fur on their head, followed by disinfection of the scalp using iodine. An incision along the midline exposed the skull, and the mice were then carefully positioned with their head secured horizontally within the stereotactic injection apparatus. This method involved targeting specific coordinates relative to the bregma. The injection site was precisely located at posterior 2.5 mm, lateral 2.0 mm, and ventral 1.7 mm. Perioperative care and anesthesia received meticulous attention during the surgical procedure. The mice were anesthetized using isoflurane. Before the incision, the surgical site was thoroughly disinfected. Crucial vital signs, including heart and respiratory rates, were carefully monitored throughout the procedure. To prevent hypothermia, the mice were maintained at an appropriate temperature. Following surgery, the mice recuperated in a quiet, sterile environment and received post-operative care to ensure their well-being during the recovery period.

### Primary neuronal culture

Primary cortical neurons were isolated from tau P301S mouse embryos at embryonic day 18 (E18). The embryos were dissected from the uterine horns and sterilized with 75% ethanol and sterile PBS. The brains were then transferred to a fresh dish with PBS, the meninges were removed, and the cortex tissue was extracted. The extracted cortex tissue was incubated with trypsin/EDTA at 37°C for 15 min and halted with DMEM-HS-FS (DMEM supplemented with horse serum and fetal serum). After centrifugation, the cell pellet was resuspended in 3 mL of DMEM-HS-FS to obtain a single-cell suspension, which was plated in 6-well plates. The primary neurons were cultured in neurobasal medium supplemented with B-27, 0.5 mM L-glutamine, penicillin, and streptomycin at 37°C in a 5% CO_2_/95% air environment. To overexpress γ-adducin in primary neurons, AAVs encoding GFP-vector, GFP-γ-adducin, and GFP-γ-adducin 1–357 fragment were introduced. After seven days, neurons were fixed with 4% PFA and subjected to immunostaining.

### Cell culture and transfection

HEK293 cells and SH-SY5Y cells were cultured in Dulbecco’s modified Eagle medium (DMEM) supplemented with 10% fetal bovine serum (FBS) and 1% penicillin–streptomycin. The cells were maintained at 37°C in a 5% CO_2_/95% air environment. To overexpress tau and γ-adducin, the cells were transfected with HA-tau plasmids along with plasmids encoding GST-vector, GST-γ-adducin, and GST-γ-adducin 1–357 fragment using polyethyleneimine (PEI). Transfection was performed with cells at a confluence of 60–70%. After 24 h, GFP expression was observed using fluorescence microscopy to evaluate transfection efficiency. 4-benzyl-2-methyl-1,2,4-thiadiazolidine-3,5-dione (TDZD-8) (MedChemExpress, Cat#HY-11012), a specific non-competitive inhibitor of GSK-3β, was dissolved in dimethyl sulfoxide (DMSO) to prepare a 20 mM stock solution. HEK293 cells and SH-SY5Y cells were transfected with HA-tau plasmids along with plasmids encoding GST-vector, GST-γ-adducin, and GST-γ-adducin 1–357 fragment for 6 h, followed by the addition of TDZD-8 solution to reach a final concentration of 20 μM TDZD-8. After 48 h of incubation, cell samples were stored at −80°C for further experimentation.

### Western blot analysis

The mice were anesthetized and perfused transcardially with saline. The brain was carefully removed from the skull. The brain was dissected to isolate the specific region of the hippocampus. Protein extracts were prepared from either cultured cells or the mouse hippocampus using lysis buffer containing a phosphatase and protease inhibitor cocktail. The protein extracts were separated on a 12% SDS–PAGE gel and transferred to nitrocellulose membranes using a semidry system. After blocking with 5% skim milk for 1 h, the membranes were incubated with primary antibodies overnight at 4°C. After undergoing three washes with Tris-buffered saline with Tween 20 (TBST), membranes were incubated with HRP-conjugated antibodies. Subsequently, the membranes underwent three additional TBST washes. Protein bands were then visualized using enhanced chemiluminescence (ECL) within a Western blotting system (Bio-Rad). The antibodies used in the Western blot experiment are listed in Table 1.

**Table 1:** Antibody information.

**Primary antibody**	**Manufacturer**	**Catalog number**	**Dilution**
Mouse monoclonal anti-GST	Proteintech	66,001-2-Ig	1:10000 for Western blot
Mouse monoclonal anti-HA	Proteintech	66,006-2-Ig	1:5000 for Western blot
Mouse monoclonal anti-GAPDH	Proteintech	60,004-1-Ig	1:8000 for Western blot
Mouse monoclonal anti-tau (Tau5)	Thermo Fisher Scientific	MA5-12808	1:2000 for Western blot
Rabbit Polyclonal anti-CDK5	Proteintech	10,430-1-AP	1:1000 for Western blot
Rabbit Polyclonal anti-P35	Proteintech	18,346-1-AP	1:1000 for Western blot
Rabbit Polyclonal anti-Akt	Cell Signaling Technology	4,691	1:1000 for Western blot
Rabbit Polyclonal anti-phospho-Akt	Cell Signaling Technology	4,060	1:1000 for Western blot
Rabbit monoclonal anti-GSK-3β (Y174)	Abcam	ab32391	1:1000 for Western blot
Rabbit Polyclonal anti-phospho-GSK-3β (Tyr216)	Proteintech	29,125-1-AP	1:1000 for Western blot
Rabbit monoclonal anti-phospho-GSK-3β (Ser9)	Cell Signaling Technology	9322S	1:1000 for Western blot
Mouse monoclonal anti-GFP	Proteintech	66,002-1-Ig	1:10000 for Western blot 1:1000 for Immunofluorescence
Rabbit monoclonal anti-phospho-Tau (Ser396)	Abcam	ab109390	1:2000 for Western blot 1:1000 for Immunofluorescence
Secondary antibody	Manufacturer	Catalog number	Dilution
Goat anti-rabbit IgG (H + L)-HRP conjugate	Bio-Rad	1,706,515	1:8000 for Western blot
Goat anti-mouse IgG (H + L)-HRP conjugate	Bio-Rad	1,706,516	1:8000 for Western blot
Goat anti-mouse IgG (H + L) Alexa Fluor™ 488	Thermo Fisher Scientific	A11001	1:1000 for Immunofluorescence
Goat anti-rabbit IgG (H + L) Alexa Fluor™ 594	Thermo Fisher Scientific	A11012	1:1000 for Immunofluorescence

### Immunofluorescence

The anesthetized mice were transcardially perfused using saline and 4% paraformaldehyde. Extracted brain tissues were dehydrated and then sliced into 4-μm sections after embedding in paraffin. Initially, the paraffin-embedded sections were treated with xylene to remove paraffin and gradually rehydrated using a series of ethanol solutions. Next, the sections underwent heat-induced antigen retrieval at 94°C for 20 min in 10 mM sodium citrate buffer (pH 6.0). After washing with phosphate-buffered saline (PBS), endogenous peroxidase activity was neutralized with a 0.3% hydrogen peroxide solution for 10 min. After blocking with 3% bovine serum albumin (BSA), the sections were incubated with primary antibodies overnight at 4°C. After a PBS rinse, goat anti-rabbit IgG (H + L) Alexa Fluor™ 594 secondary antibodies were applied for 2 h at room temperature. Nuclei were stained with 4′,6-diamidino-2-phenylindole (DAPI). Finally, fluorescence images were captured using a fluorescence microscope from Olympus.

For immunocytofluorescence, cultured cells were initially fixed and permeabilized using a solution of 4% paraformaldehyde and 1% Triton X-100 for 10 min. Subsequently, the slides were washed with PBS, followed by blocking with 3% BSA for 30 min. The cells were then incubated with primary antibodies overnight at 4°C. After another round of washing with PBS, the cells were exposed to goat anti-mouse IgG (H + L) Alexa Fluor™ 488 and goat anti-rabbit IgG (H + L) Alexa Fluor™ 594 secondary antibodies for 2 h at room temperature in the dark. Then, the cells were washed with PBS and subjected to nuclear staining with DAPI. After a quick rinse in PBS, the cells were examined using a fluorescence microscope from Olympus.

### Statistical analysis

Statistical analyses were conducted using GraphPad Prism (version 8.0). All data were presented as means ± standard deviation (SD) from three or more independent experiments. One-way analysis of variance (ANOVA) was performed to determine significant main effects and differences among three or more groups, followed by Tukey’s or least significant difference (LSD) multiple comparisons for *post hoc* tests. Differences with *p* values less than 0.05 were considered significant.

## Results

### The γ-adducin 1–357 fragment promotes tau phosphorylation *in vitro*

**Figure 1:**
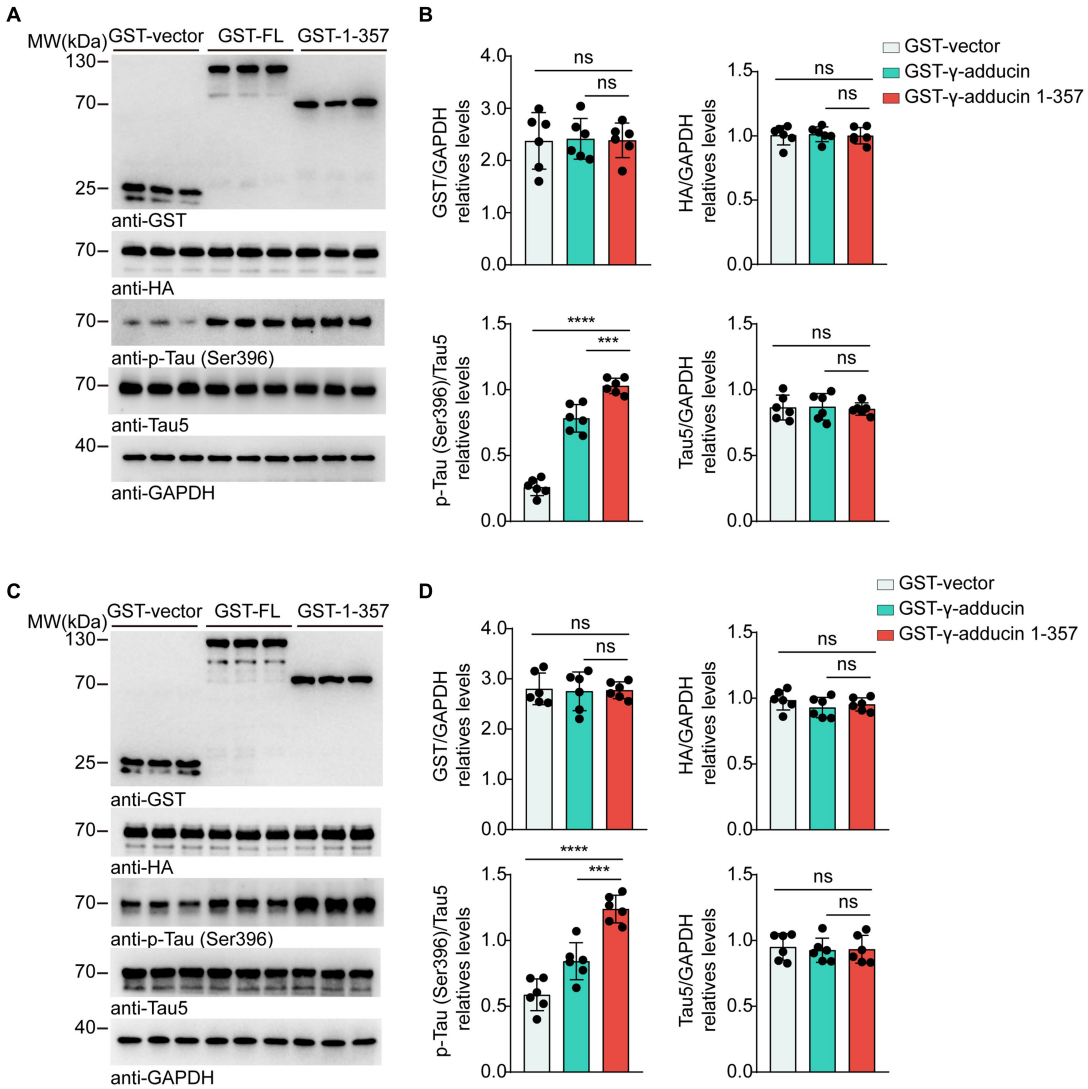
γ-Adducin 1–357 enhances tau phosphorylation *in vitro*. **(A,B)** Western blot analysis of tau phosphorylation at Ser396 in HEK293 cells co-transfected with HA-tau and GST-vector, GST-γ-adducin, or GST-γ-adducin 1–357. **(C,D)** Western blot analysis of tau phosphorylation at Ser396 in SH-SY5Y cells. Data were presented as mean ± SD, *n* = 6 independent experiments. n.s. not significant, ***p < 0.001, ****p < 0.0001, by one-way ANOVA.

To investigate whether the γ-adducin 1–357 fragment regulates tau phosphorylation in AD, we co-transfected HA-tau 1–441 together with GST-vector, GST-γ-adducin, or GST-γ-adducin 1–357 fragment into HEK293 and SH-SY5Y cells. The serine residue at position 396 (Ser396) of tau is considered the earliest phosphorylation site associated with AD (Mondragón-Rodríguez et al., 2014). Therefore, we examined the phosphorylation of S396. Subsequent analysis included calculating *F*-values, degrees of freedom (DFn, DFd), and probabilities. Western blot analysis showed that GST-γ-adducin, GST-γ-adducin 1–357 fragment, and HA-tau were overexpressed after transfection. Interestingly, Western blot analysis revealed that the phosphorylation of tau at Ser396 in cells overexpressing the GST-γ-adducin 1–357 fragment was higher than that in cells expressing GST-vector and GST-γ-adducin [p-Tau-Ser396 (*F* (2, 15) = 150.8, *p* < 0.0001)] (Figures 1A,B). Similar results were found in SH-SY5Y cells [p-Tau-Ser396 (*F* (2, 15) = 42.88, *p* < 0.0001)] (Figures 1C,D). These results indicate that overexpression of the γ-adducin 1–357 fragment enhances tau phosphorylation.

### The γ-adducin 1–357 fragment promotes tau phosphorylation in primary neurons

**Figure 2:**
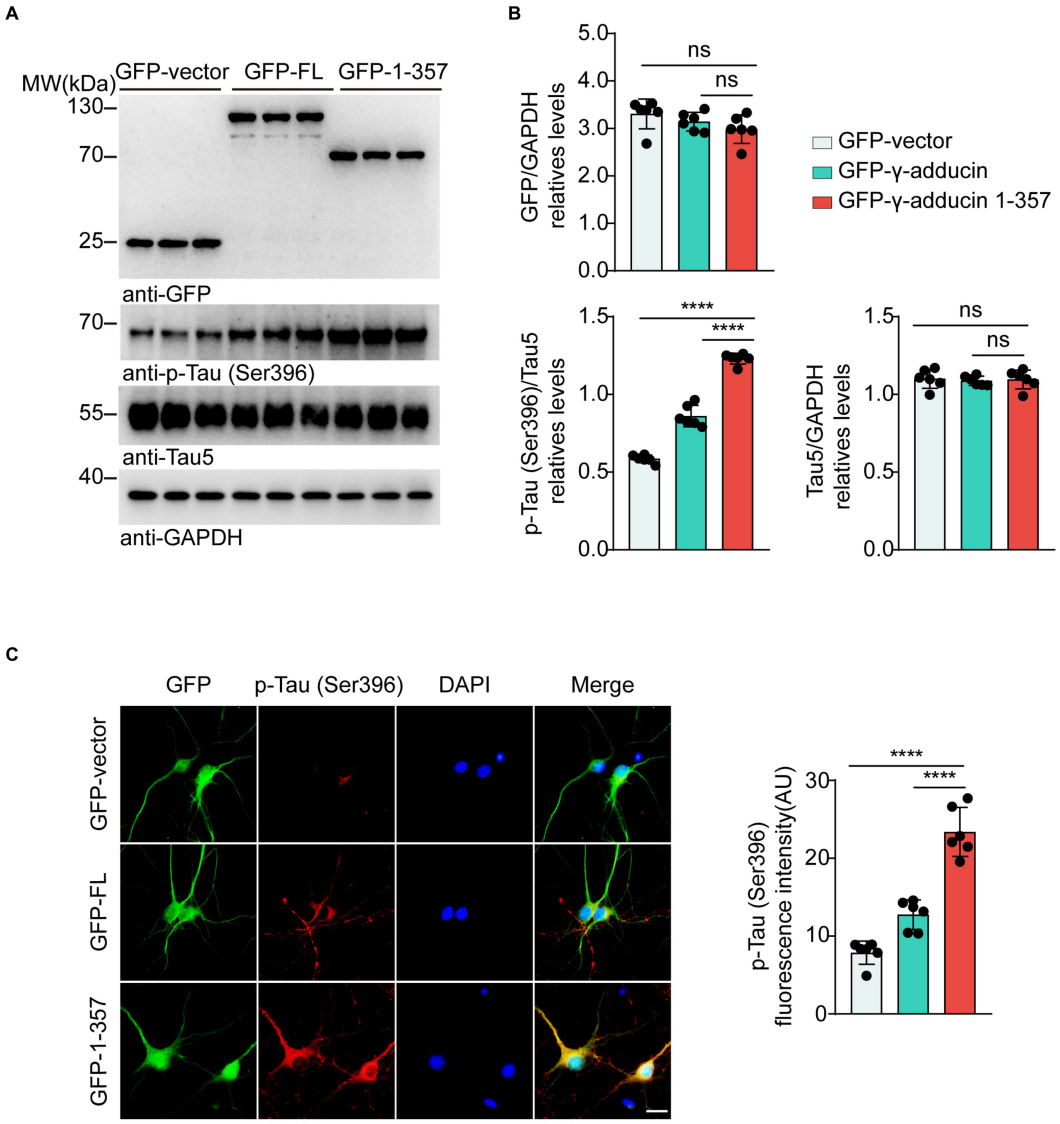
γ-Adducin 1–357 promotes tau phosphorylation in primary neurons. **(A,B)** Western blot analysis of tau phosphorylation at Ser396 in primary neurons infected with AAV-GFP, AAV-GFP-γ-adducin, and AAV-GFP-γ-adducin 1–357. Data were presented as mean ± SD, *n* = 6 independent experiments. n.s. not significant, ****p < 0.0001 by one-way ANOVA. **(C)** Immunofluorescence of phosphorylated tau (red) along with GFP-tag (green) in primary neurons. Scale bar, 20 μm. Data were presented as mean ± SD, n = 6 independent experiments. ****p < 0.0001. AU, arbitrary units.

To explore the effect of the γ-adducin 1–357 fragment on tau phosphorylation in primary neurons, we infected neurons with AAVs encoding GFP-vector, GFP-γ-adducin, or GFP-γ-adducin 1–357 fragment. Western blot analysis showed that GFP-γ-adducin and the GFP-γ-adducin 1–357 fragment were efficiently expressed. The levels of tau phosphorylated at Ser396 were higher in neurons infected with the GFP-γ-adducin 1–357 fragment than in neurons infected with AAV-GFP and AAV-γ-adducin, while the levels of total tau remained unchanged [p-Tau-Ser396 (*F* (2, 15) = 287.7, *p* < 0.0001)] (Figures 2A,B). These results were confirmed by immunofluorescence staining [p-Tau-Ser396 (*F* (2, 15) = 71.86, *p* < 0.0001)] (Figure 2C). Thus, the γ-adducin 1–357 fragment enhances tau phosphorylation in primary neurons.

### GSK-3β mediates tau phosphorylation induced by γ-adducin 1–357

**Figure 3:**
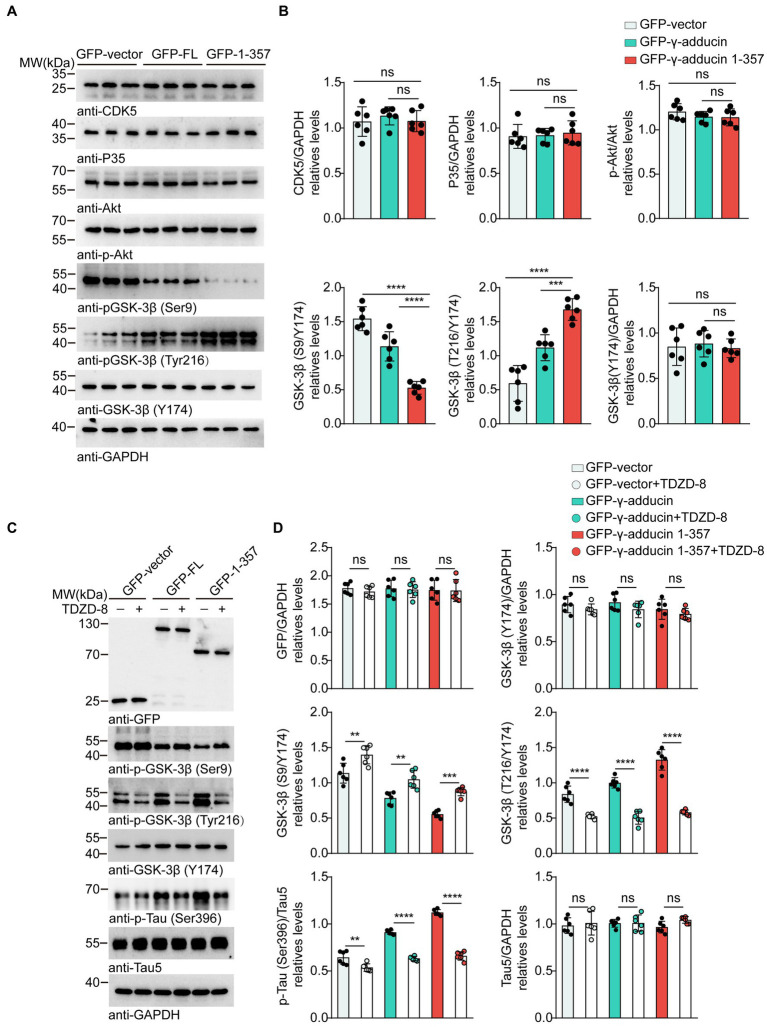
GSK-3β mediates tau phosphorylation induced by γ-adducin 1–357. **(A,B)** Western blot of the levels of CDK5 (Cyclin-dependent kinase 5), Akt (Protein kinase B), and GSK-3β (Glycogen synthase kinase-3β) in primary neurons. **(C,D)** Western blot analysis of the levels of GSK3β, p-tau Ser396 (phosphorylated tau at Ser396), and total tau in primary neurons with or without TDZD-8 (specific kinase inhibitor TDZD-8). Data were presented as mean ± SD, n = 6 independent experiments. n.s. not significant, **p < 0.01, ***p < 0.001, ****p < 0.0001 by one-way ANOVA.

Multiple kinases contribute to tau phosphorylation. Cyclin-dependent kinase 5 (CDK5), protein kinase B (AKT), and glycogen synthase kinase-3β (GSK-3β) are the most well-studied tau kinases. To identify the kinases involved in γ-adducin 1-357-induced tau phosphorylation, we infected primary neurons with the AAV-GFP-γ-adducin 1–357 fragment and determined the activity of CDK5, AKT, and GSK-3β(Lauretti et al., 1867; Kumar and Bansal, 2022; Maitra and Vincent, 2022). Western blot analysis revealed that the phosphorylation of GSK-3β at Ser9, which negatively regulates GSK-3β activity, was decreased, while the phosphorylation of GSK-3β at Tyr216, which positively activates GSK-3β, was increased. The levels of CDK5 and AKT were not altered in neurons overexpressing γ-adducin 1–357 [p-GSK-3β-Ser9 (*F* (2, 15) = 54.66, *p* < 0.0001] and p-GSK-3β-Tyr216 [*F* (2, 15) = 40.62, *p* < 0.0001)] (Figures 3A,B). To ascertain the role of GSK-3β in tau phosphorylation induced by γ-adducin 1–357, neurons were treated with TDZD-8, a specific GSK-3β inhibitor (Koehler et al., 2019; Lei et al., 2019). We observed decreased levels of p-GSK3β (Tyr216), along with a reduction in the phosphorylation of tau at Ser396 in neurons (p-GSK-3β-Ser9 [*F* (5, 30) = 47.76, *p* < 0.0001] and p-GSK-3β-Tyr216 [F (5, 30) = 75.71, *p* < 0.0001)] (Figures 3C,D). These findings provide further evidence supporting the specific role of GSK-3β in promoting tau phosphorylation induced by γ-adducin 1–357.

### γ-Adducin 1–357 fragment enhances tau phosphorylation in a mouse model of AD

**Figure 4:**
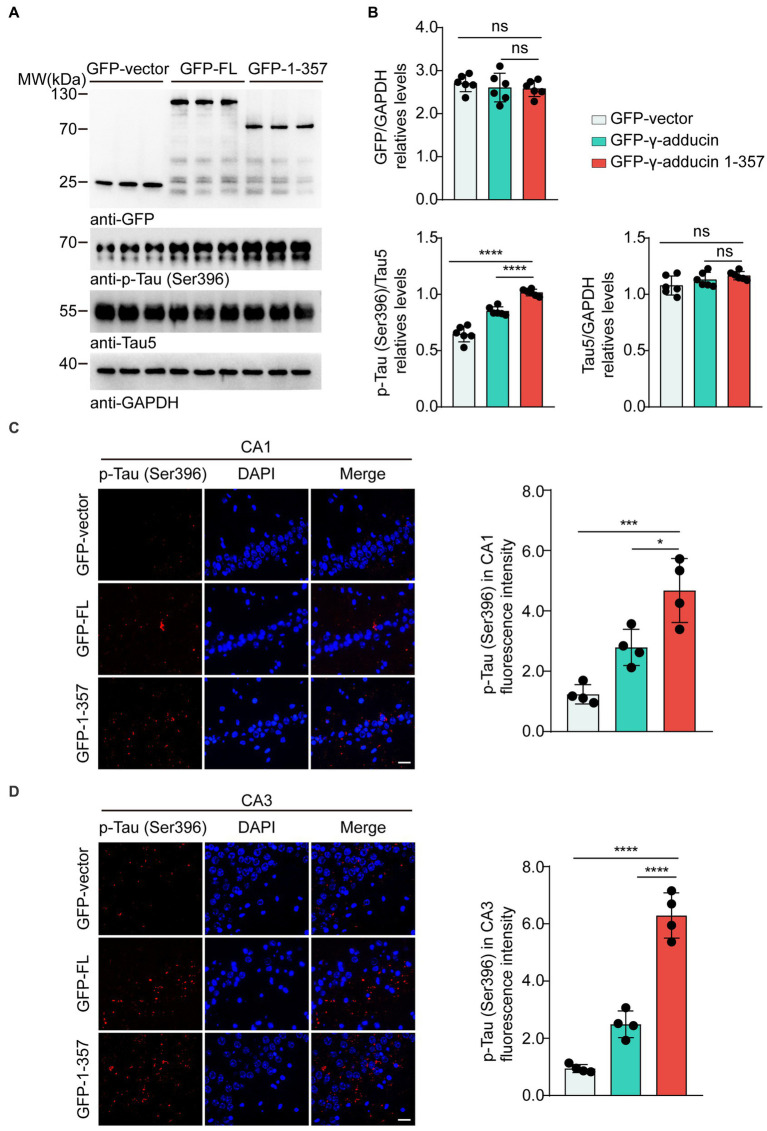
γ-Adducin 1–357 enhances tau phosphorylation in a mouse model of AD. **(A,B)** Western blot analysis of phosphorylated tau at Ser396 in 6 month-old tau P301S mice injected with AAV-EGFP, AAV-EGFP-γ-adducin, and AAV-EGFP-γ-adducin 1–357. Data were presented as mean ± SD, n = 6 mice per group. n.s. not significant, ****p < 0.0001 by one-way ANOVA. **(C,D)** Immunofluorescence of phosphorylated tau (red) with DAPI staining (blue) in various brain regions. Scale bar, 20 μm. Data were presented as mean ± SD, *n* = 4 mice per group. *p < 0.05, ***p < 0.001, ****p < 0.0001. AU, arbitrary units.

To investigate whether the γ-adducin 1–357 fragment promotes tau phosphorylation *in vivo*, we performed intrahippocampal injections of AAV-GFP, AAV-GFP-γ-adducin, and AAV-GFP-γ-adducin 1–357 fragment in 2-month-old tau P301S mice. The phosphorylation of tau was determined using Western blotting at 4 months after injection. We found a dramatic increase in the presence of p-tau at Ser396 in the hippocampal sections where the γ-adducin 1–357 fragment was overexpressed. However, the levels of total tau remained unchanged [p-Tau-Ser396 (*F* (2, 15) = 84.98, *p* < 0.0001)] (Figures 4A,B). Furthermore, immunofluorescence analysis of brain slices showed enhanced p-tau Ser396 immunoreactivity in the hippocampus of mouse brains overexpressing γ-adducin 1–357 [CA1 (*F* (2, 9) = 22.44, *p* = 0.0003) and CA3 (*F* (2, 9) = 105.8, *p* < 0.0001)] (Figures 4C,D).

## Discussion

Over phosphorylation and aggregation of Tau are widely considered pathological hallmarks in AD (Wegmann et al., 2021). The factors initiating tau phosphorylation in the AD brain remain unknown. Thus, tau phosphorylation has been extensively researched. Here we focused on the effect of the γ-adducin 1–357 fragment on tau phosphorylation. We found that the γ-adducin 1–357 fragment promotes the phosphorylation of tau at Ser396, which is one of the early pathological signs of AD (Mondragón-Rodríguez et al., 2014). Furthermore, the activation of GSK-3β by the γ-adducin 1–357 fragment underscores its role as a critical kinase involved in tau pathology. The inhibition of GSK-3β activity by the specific kinase inhibitor TDZD-8 suggests the possibility of targeted therapeutic interventions to modulate tau phosphorylation and its associated pathological consequences.

Previous studies have indicated that the γ-adducin 1–357 fragment induces synaptic dysfunction and promotes cognitive impairments in AD. The γ-adducin 1–357 fragment inhibits neurite outgrowth by disrupting spectrin-actin assembly (Xiong et al., 2021). Building upon this foundation, our current study demonstrates that γ-adducin 1–357 fragment enhances tau phosphorylation. It has been reported that over phosphorylation of tau also affects neurite outgrowth (Morris and Brady, 2022). Thus, γ-adducin 1–357 fragment may not only directly influence the formation of actin filaments, but also interfere with neurite outgrowth by enhancing tau phosphorylation.

We demonstrate that the upregulation of the γ-adducin 1–357 fragment triggers tau phosphorylation by activating GSK-3β. Targeting the aberrant phosphorylation of tau holds promise for potential clinical therapies. GSK3-β is considered a key player in AD pathophysiology since dysregulation of this kinase influences the major hallmarks of the disease, including tau phosphorylation, amyloid-β production, memory, neurogenesis, and synaptic function(Lauretti et al., 1867). Recent decades have witnessed concerted efforts to comprehend the role of GSK-3β, with the aim of designing potent and selective GSK-3β inhibitors. However, clinical and preclinical trials using GSK-3β inhibitors have exhibited suboptimal potency (Llorens-Martín et al., 2014; Xia et al., 2021). Likewise, a combinatorial approach involving multiple drugs may be necessary to counteract tau phosphorylation, given the involvement of numerous kinases that target multiple phosphorylation sites.

Upon cleavage of γ-adducin to generate the 1–357 fragment, its actin-binding activity is compromised, leading to disruption of the cellular cytoskeleton (Xu et al., 2013). This structural alteration in the γ-adducin 1–357 fragment may expose specific regions or functional motifs, enhancing its interaction with GSK-3β and tau. Moreover, the γ-adducin 1–357 fragment might disrupt the regulatory mechanisms controlling GSK-3β activity and its interactions with downstream substrates like tau. In contrast to full-length γ-adducin, the γ-adducin 1–357 fragment exhibits enhanced activity in enhancing tau Ser396 phosphorylation and regulating GSK-3β Ser9 and Tyr216 phosphorylation. This phenomenon could be ascribed to underlying structural changes, altered binding affinity, and the potential disturbance of regulatory pathways.

The current findings lay a foundation for future investigations into the mechanisms and therapeutic strategies targeting the γ-adducin 1–357 fragment and GSK-3β in AD. However, it remains unknown how the γ-adducin 1–357 fragment activates GSK-3β. Subsequent studies could delve into the molecular interactions, signaling pathways, and efficacy of targeted interventions aimed at mitigating tau phosphorylation and its linked pathological consequences.

## Data availability statement

The original contributions presented in the study are included in the article/Supplementary material, further inquiries can be directed to the corresponding authors.

## Ethics statement

The animal study was approved by All experimental procedures were approved by the Institutional Animal Care and Use Committee (IACUC) of Renmin Hospital, Wuhan University (IACUC issue number: WDRM animal [welfare] 202200084). The study was conducted in accordance with the local legislation and institutional requirements.

## Author contributions

ZZ and LM conceived the project. HY performed most of the experiments and analyzed the data. MX contributed to the design of the methodology. CL and DX helped with cell culture. All authors contributed to the article and approved the submitted version.

## Funding

This work was supported by grants from the National Key Research and Development Program of China (2019YFE0115900), the National Natural Science Foundation of China (Nos. 82271447 and 81901090), the Innovative Research Groups of Hubei Province (2022CFA026), and and the “New 20 Terms of Universities in Jinan” grant (No. 202228022).

## Conflict of interest

The authors declare that the research was conducted in the absence of any commercial or financial relationships that could be construed as a potential conflict of interest.

## Publisher’s note

All claims expressed in this article are solely those of the authors and do not necessarily represent those of their affiliated organizations, or those of the publisher, the editors and the reviewers. Any product that may be evaluated in this article, or claim that may be made by its manufacturer, is not guaranteed or endorsed by the publisher.

## Glossary

AD: Alzheimer’s disease
NFTs: Neurofibrillary tangles
MAP: Microtubule-associated protein
AEP: Aspartic Endopeptidase
TDZD-8: 4-Benzyl-2-methyl-1,2,4-thiadiazolidine-3,5-dione
ADD1: α-Adducin
ADD2: β-Adducin
ADD3: γ-Adducin
GFP: Green Fluorescent Protein
GST: Glutathione S-Transferase
HA: Hemagglutinin
AAV: Adeno-associated virus
CO_2_: Carbon Dioxide
DMS: Dimethyl sulfoxide
PBS: Phosphate-buffered saline
BSA: Bovine Serum Albumin
PFA: Paraformaldehyde
PEI: Polyethyleneimine
DAPI: 4′,6-diamidino-2-phenylindole
SDS–PAGE: sodium dodecyl sulfate-polyacrylamide gel electrophoresis
GSK-3β: glycogen synthase kinase-3β
CDK5: cyclin-dependent kinase 5
AKT: Protein kinase B
ECL: Enhanced Chemiluminescence
ANOVA: Analysis of Variance
SD: Standard Deviation
LSD: Least Significant Difference
SPSS: Statistical Package for the Social Sciences
