# Supplementary material for: The γ-Adducin 1–357 fragment promotes tau pathology
Source: Front Aging Neurosci. 2023 Sep 13;15:1241750. doi: 10.3389/fnagi.2023.1241750 (PMC10526357; doi:10.3389/fnagi.2023.1241750)

Figure 1, A

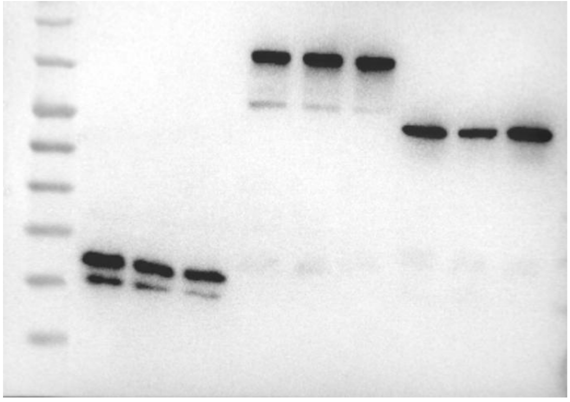

Anti-GST

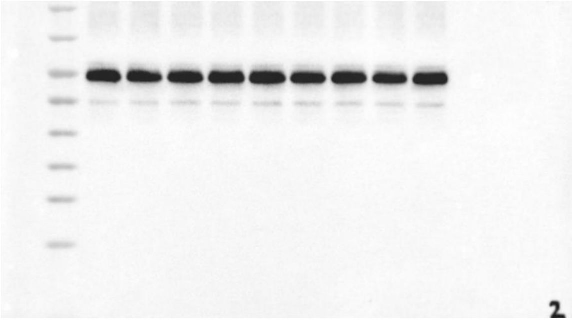

Anti-HA

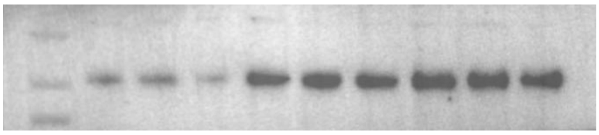

Anti-p-tau (Ser396)

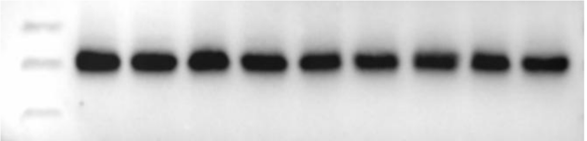

Anti-Tau5

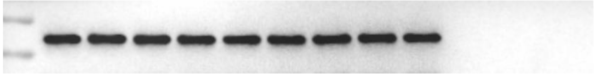

Anti-GAPDH

Figure 1, C

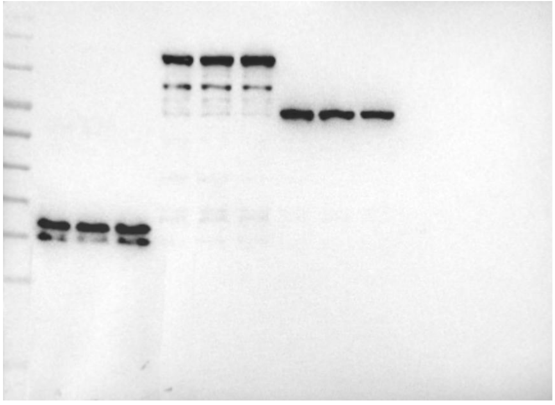

Anti-GST

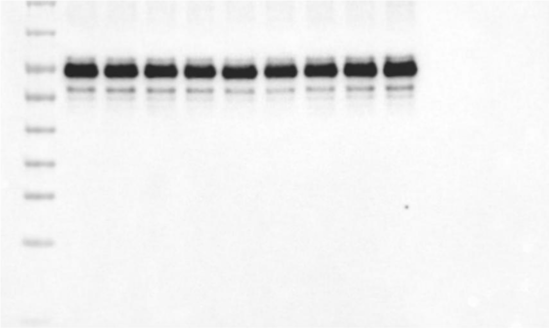

Anti-HA

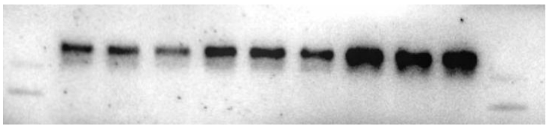

Anti-p-tau (Ser396)

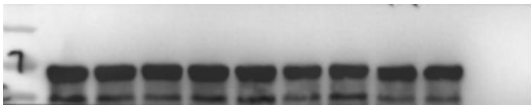

Anti-Tau5

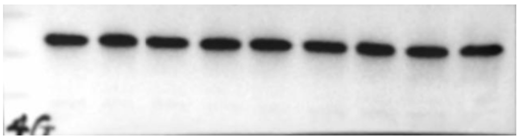

Anti-GAPDH

Figure 2, A

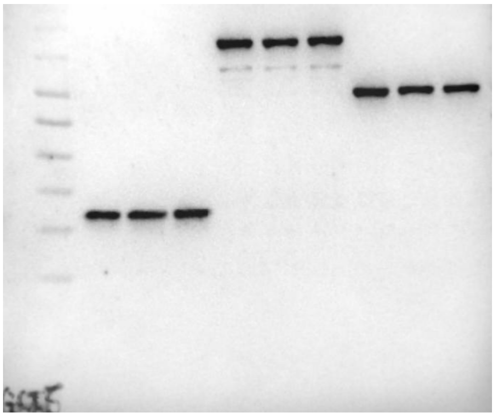

Anti-GFP

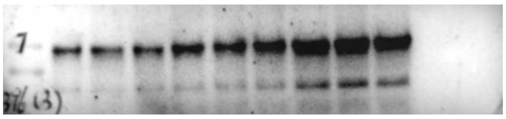

Anti-p-tau (Ser396)

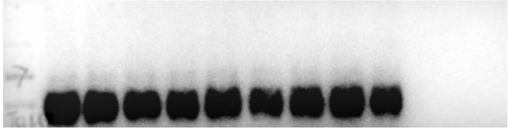

Anti-Tau5

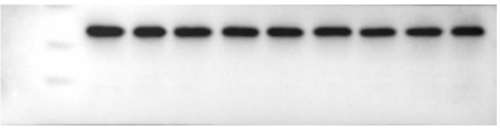

Anti-GAPDH

Figure 2, C

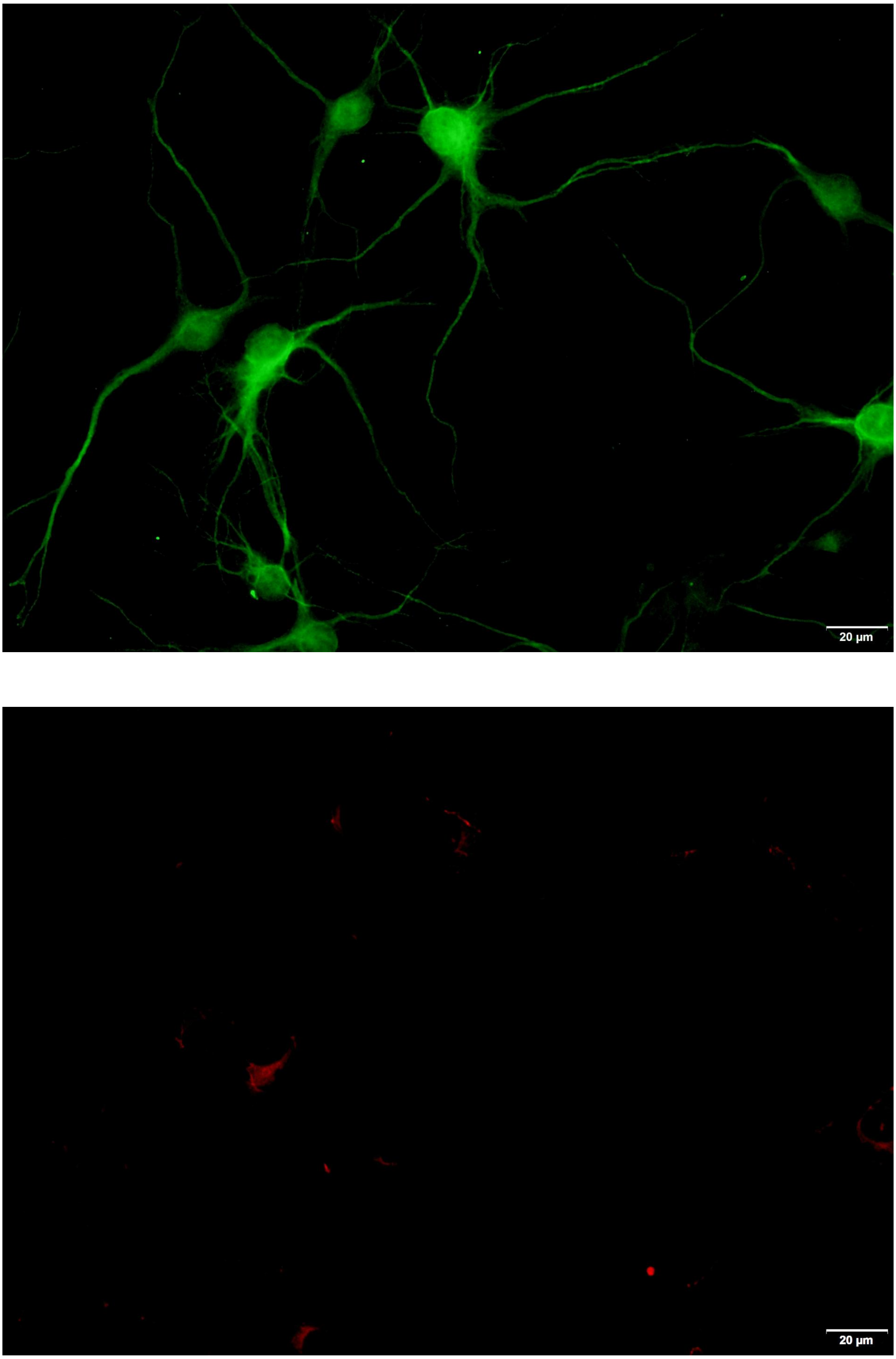

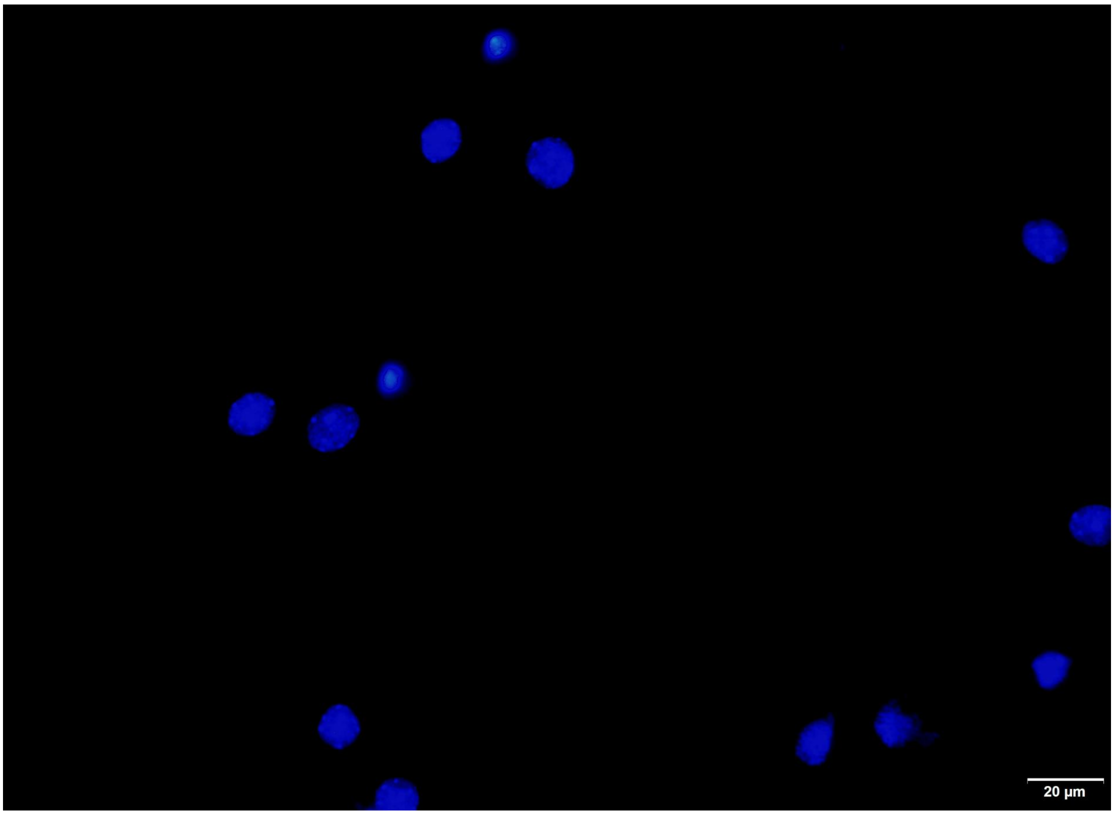
20 µm

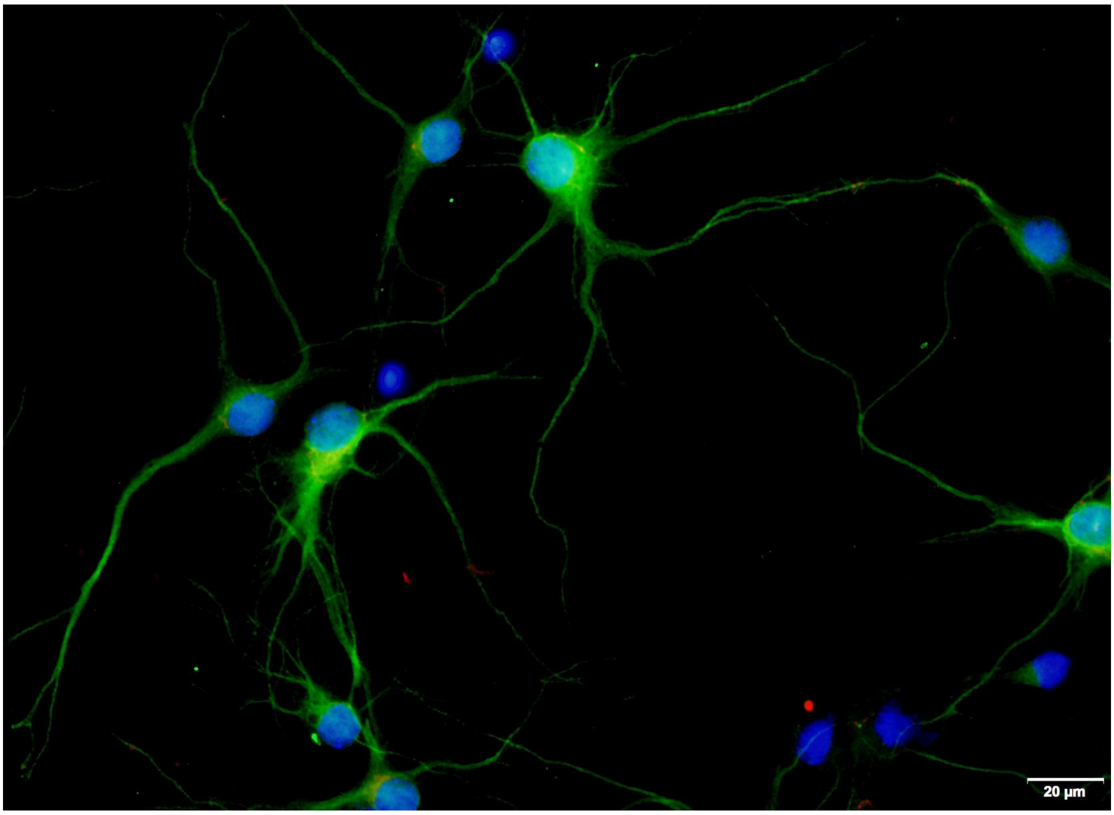
20 µm

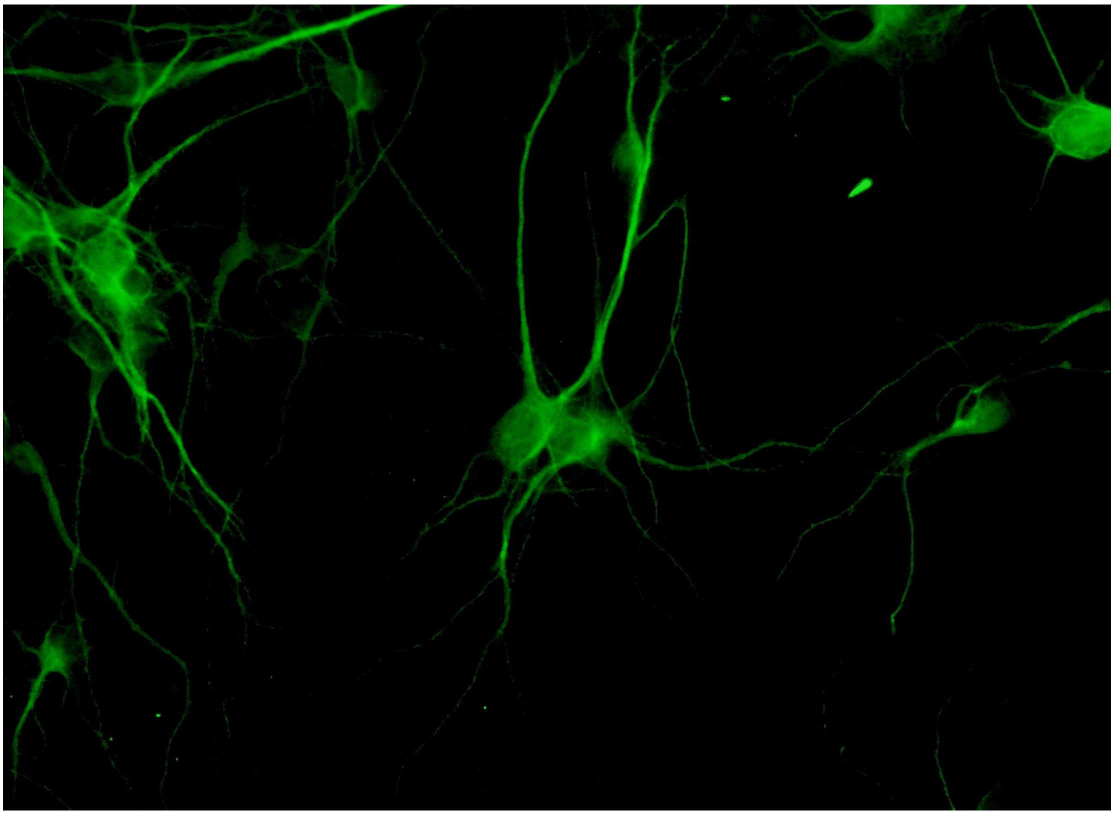

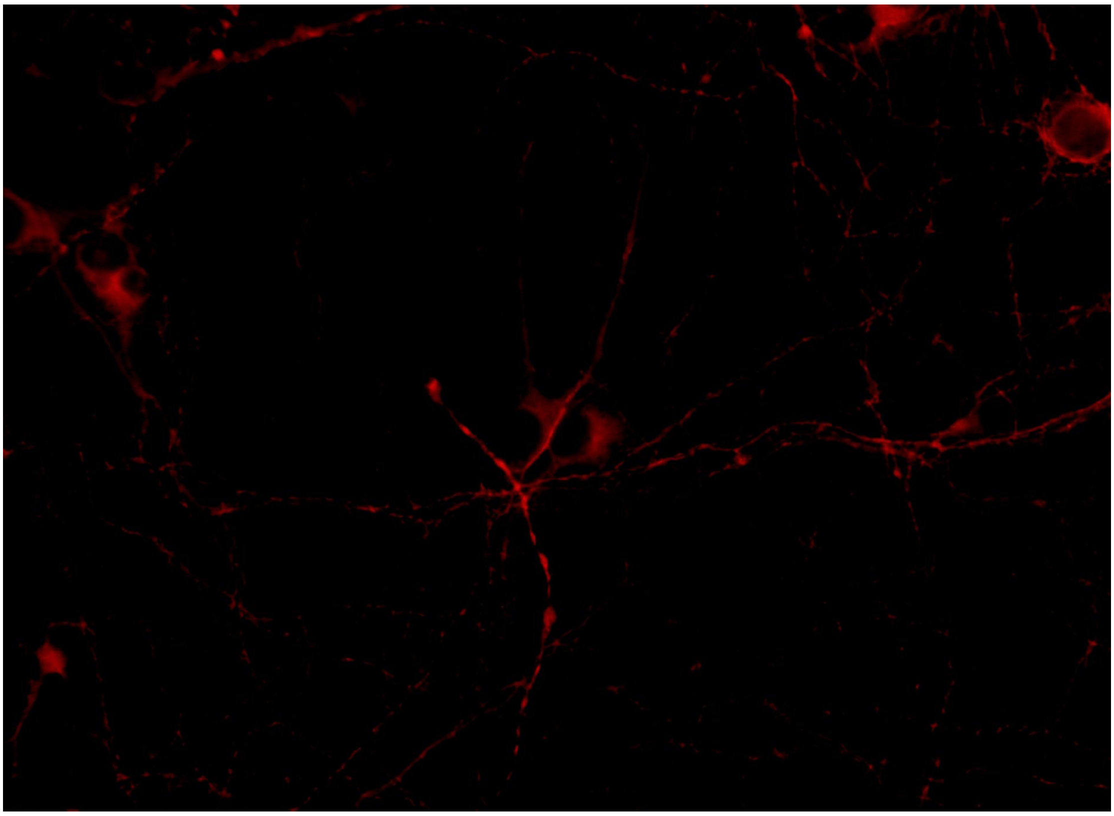

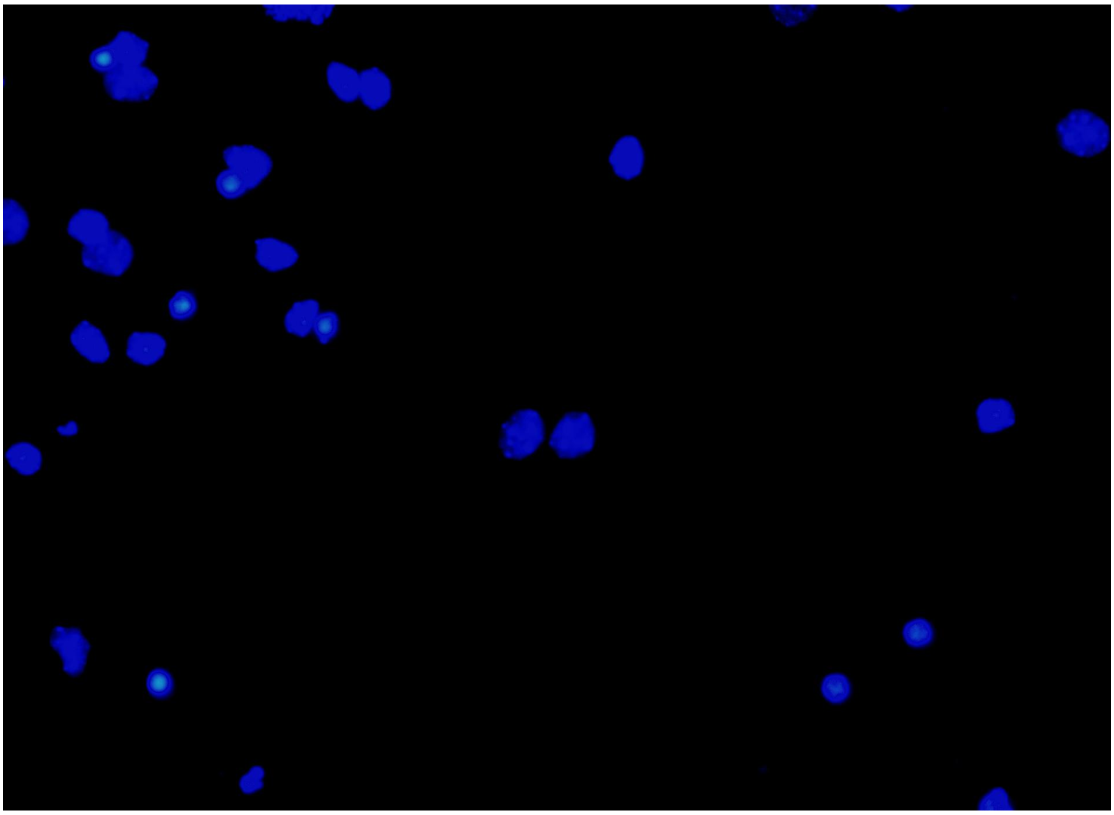

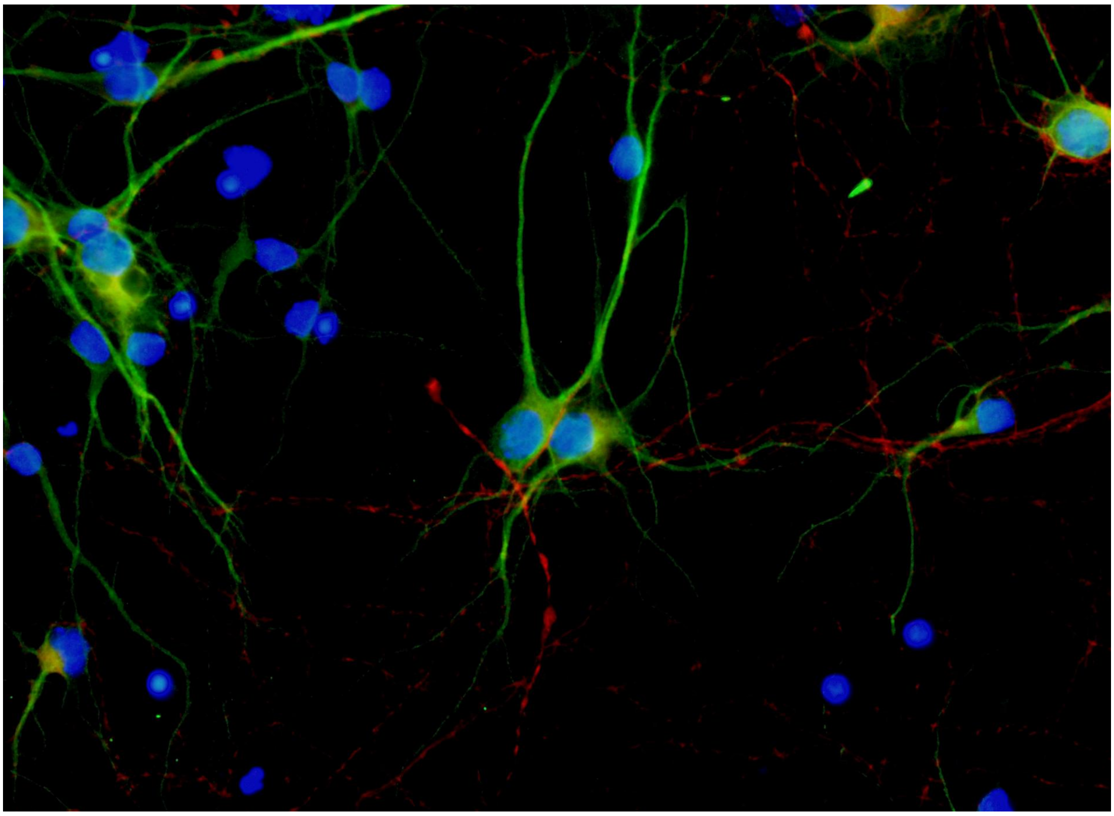

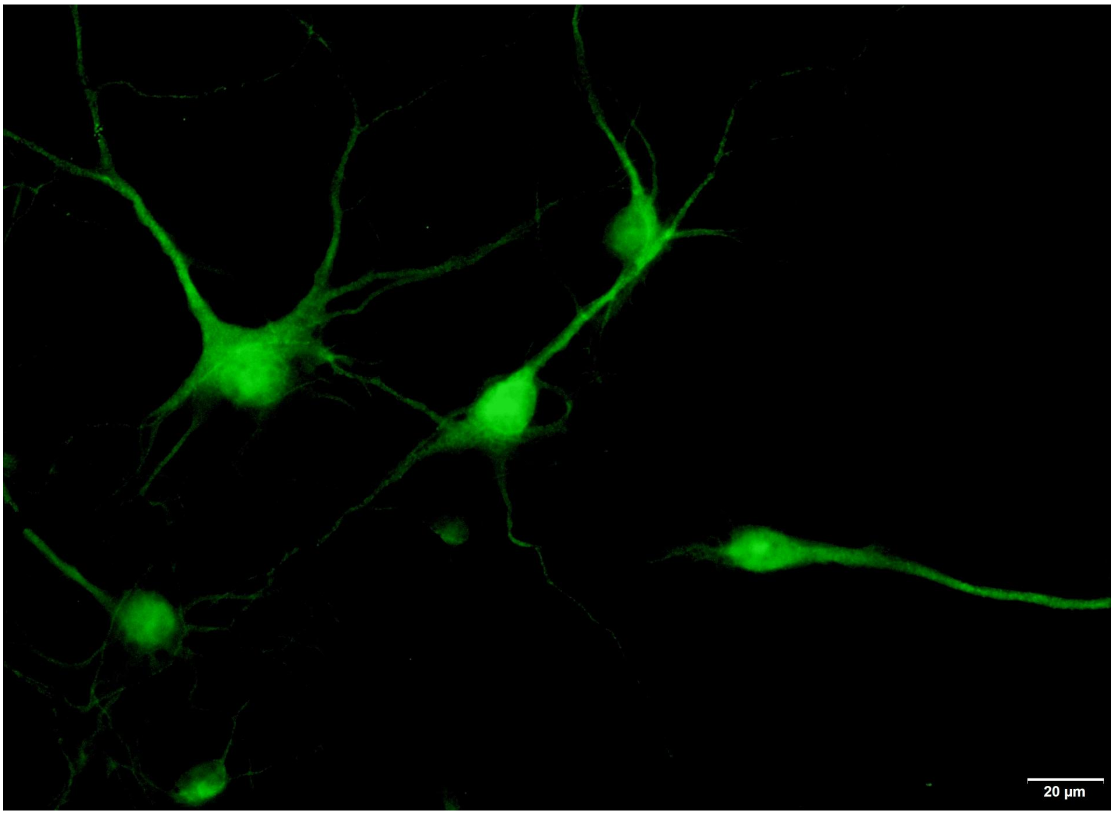
20 µm

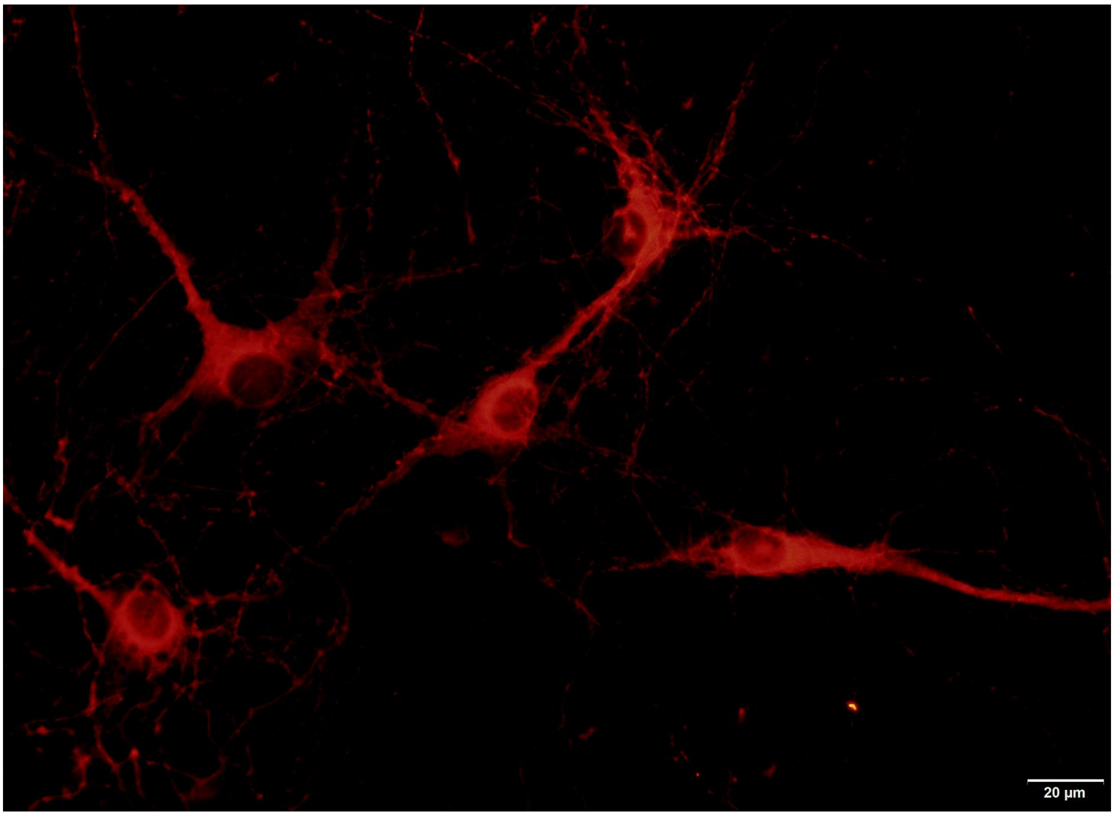
20 µm

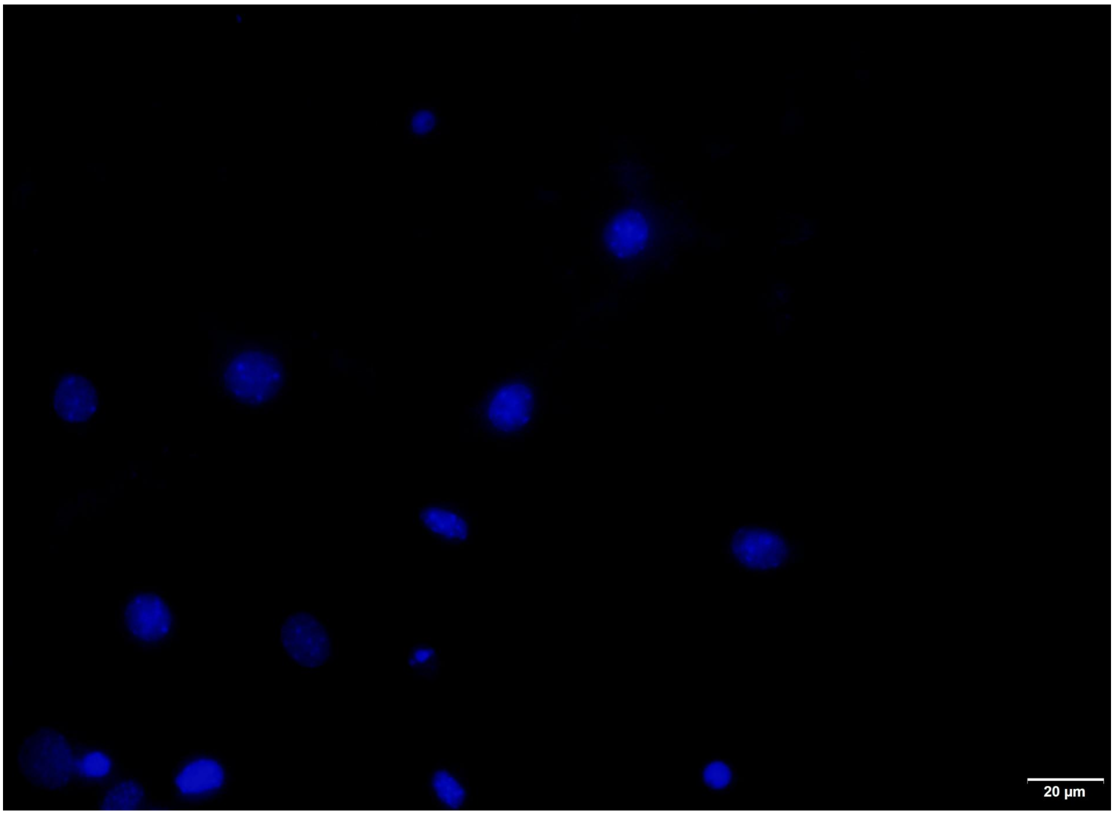
20 µm

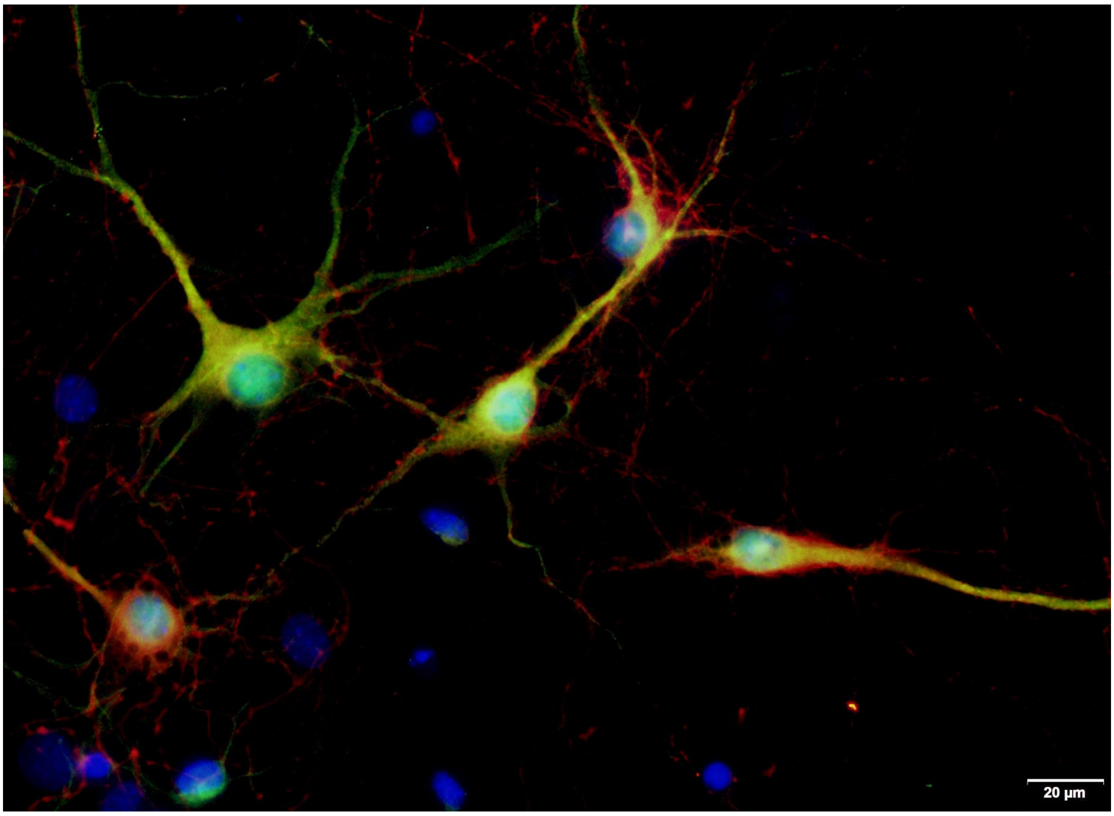
20 µm

Figure 3, A

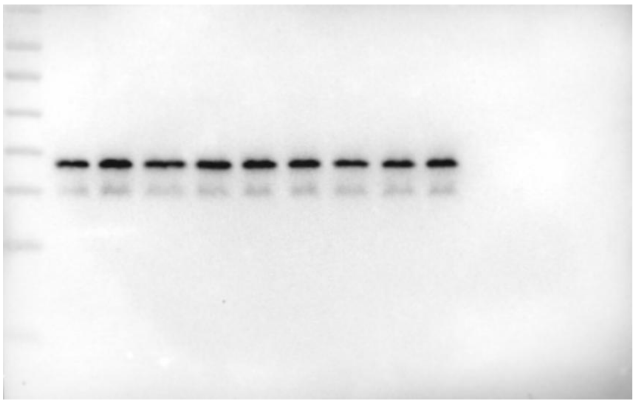

Anti-CDK5

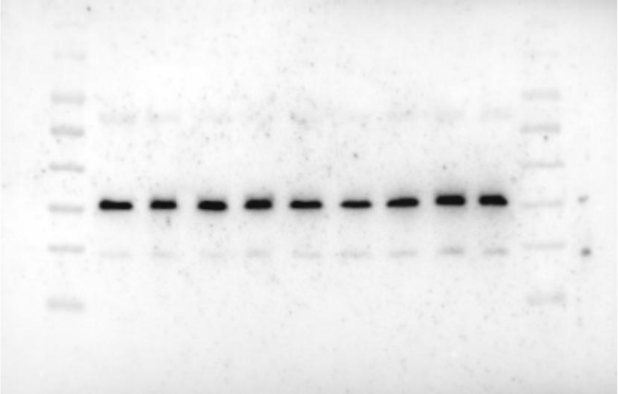

Anti-P35

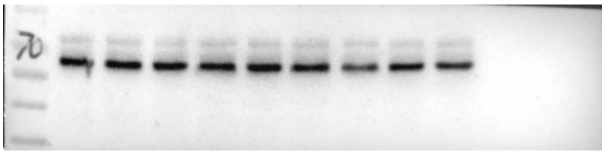

Anti-Akt

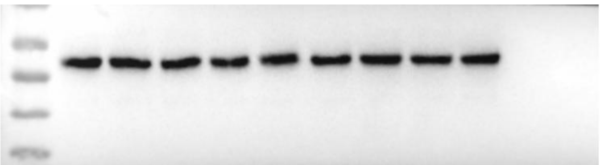

Anti-pAkt

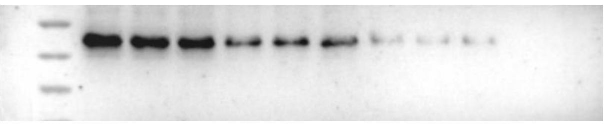

Anti-pGSK3β(Ser9)

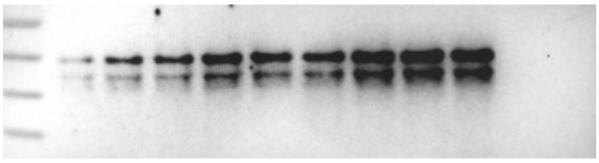

Anti-pGSK3β(Thr216)

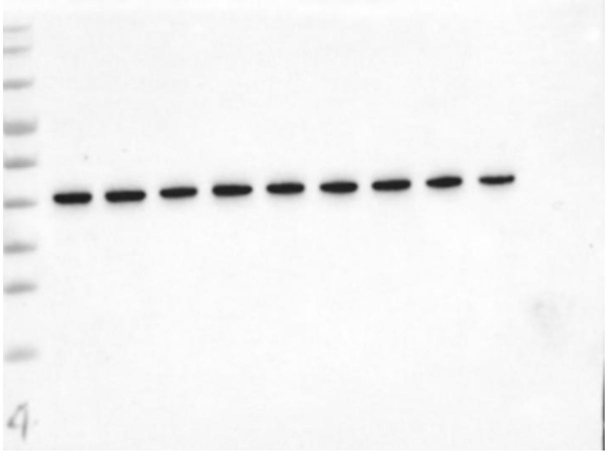

Anti-pGSK3β(Y174)

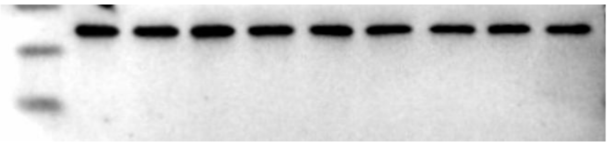

Anti-GAPDH

Figure 3, C

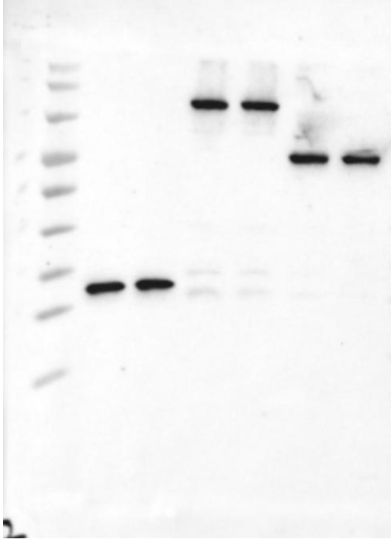

Anti-GFP

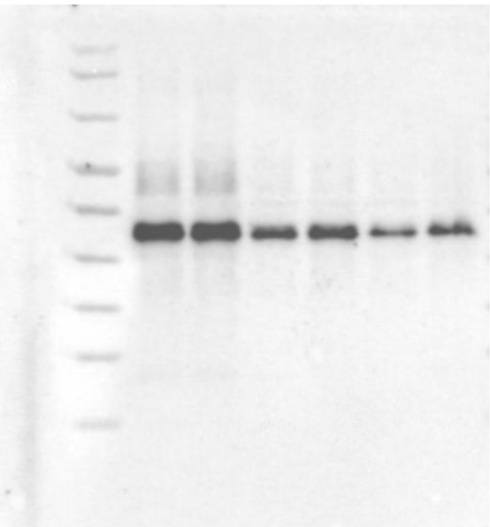

Anti-pGSK3β(Ser9)

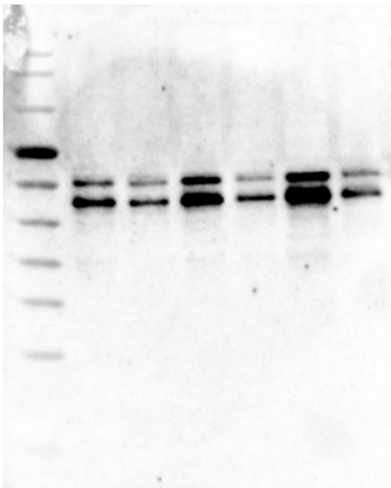

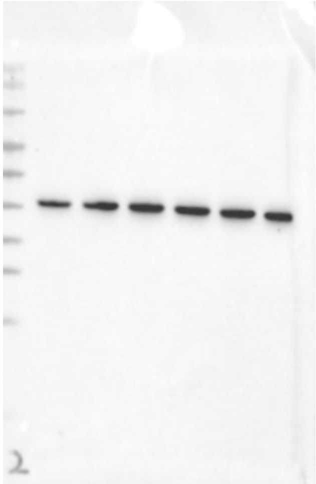

Anti-pGSK3β(Thr216)

Anti-pGSK3β(Y174)

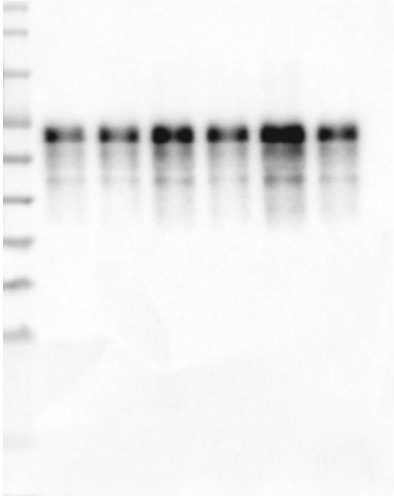

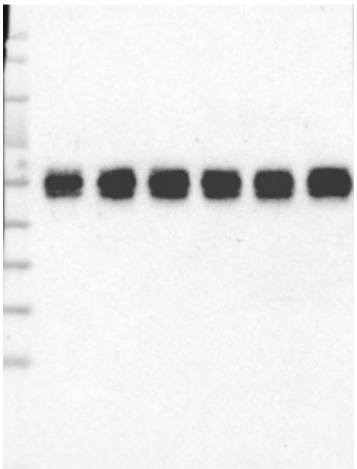

Anti-p-tau (Ser396)

Anti-Tau5

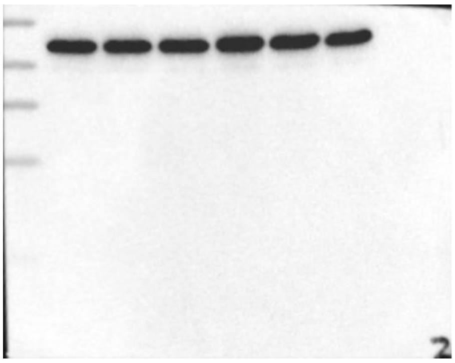

Anti-GAPDH

Figure 4, A

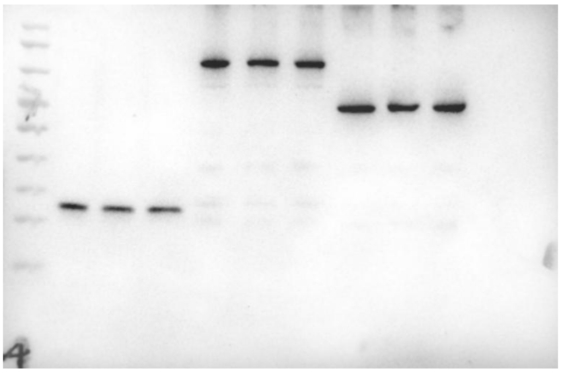

Anti-GFP

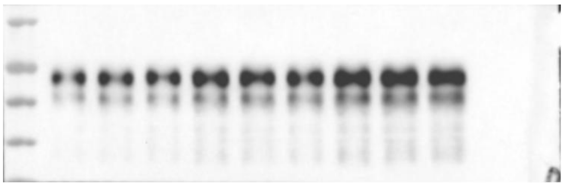

Anti-p-tau (Ser396)

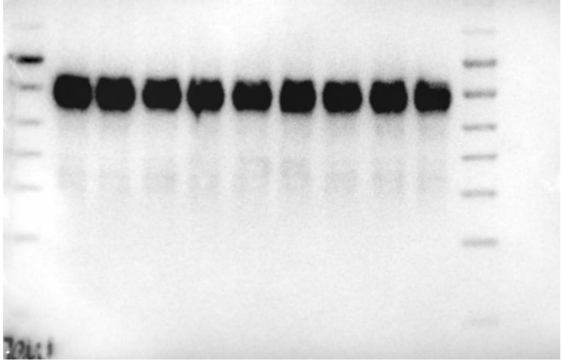

Anti-Tau5

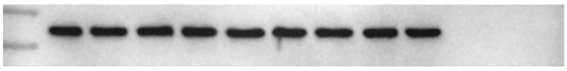

Anti-GAPDH

Figure 4,
C

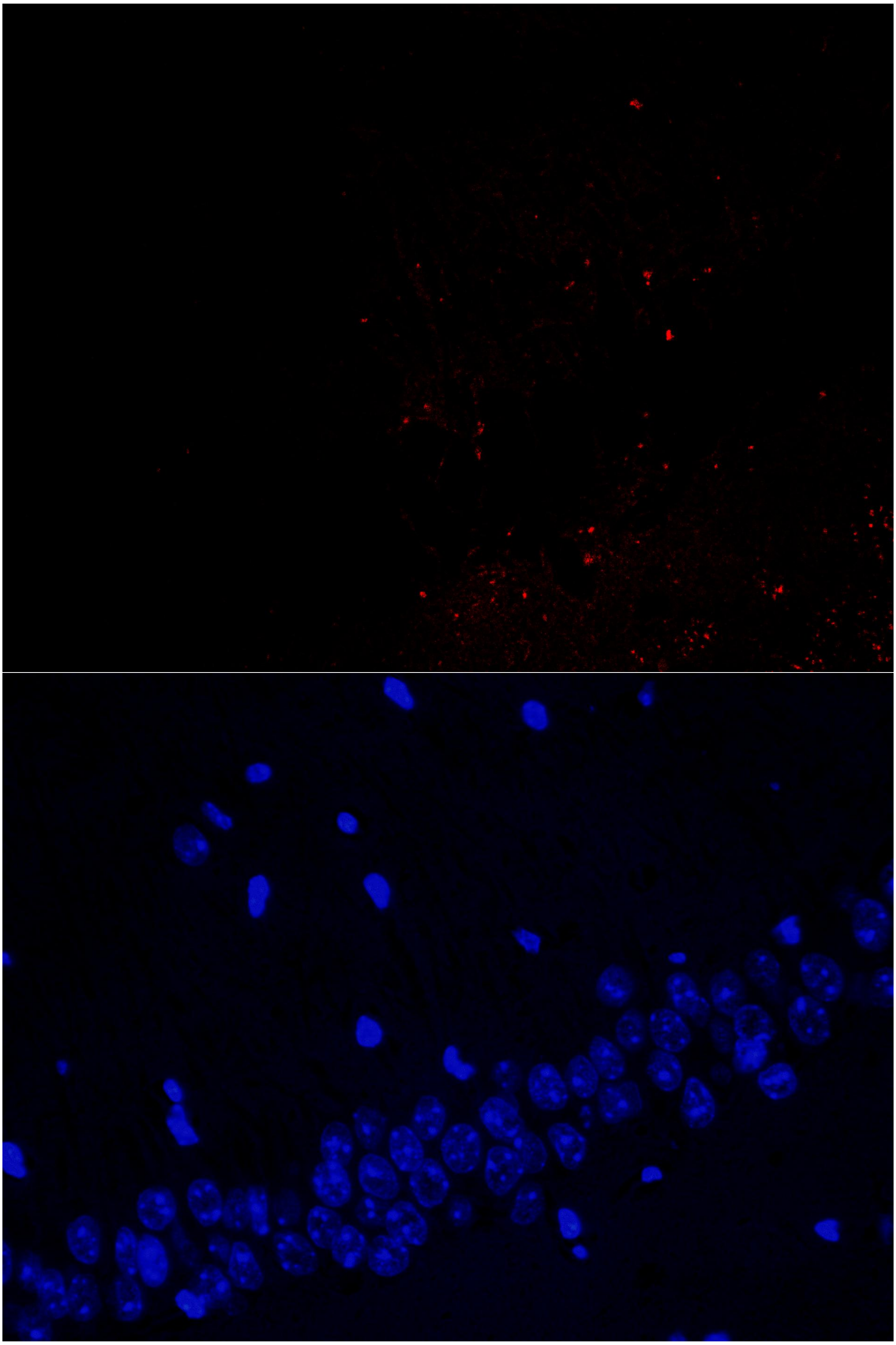

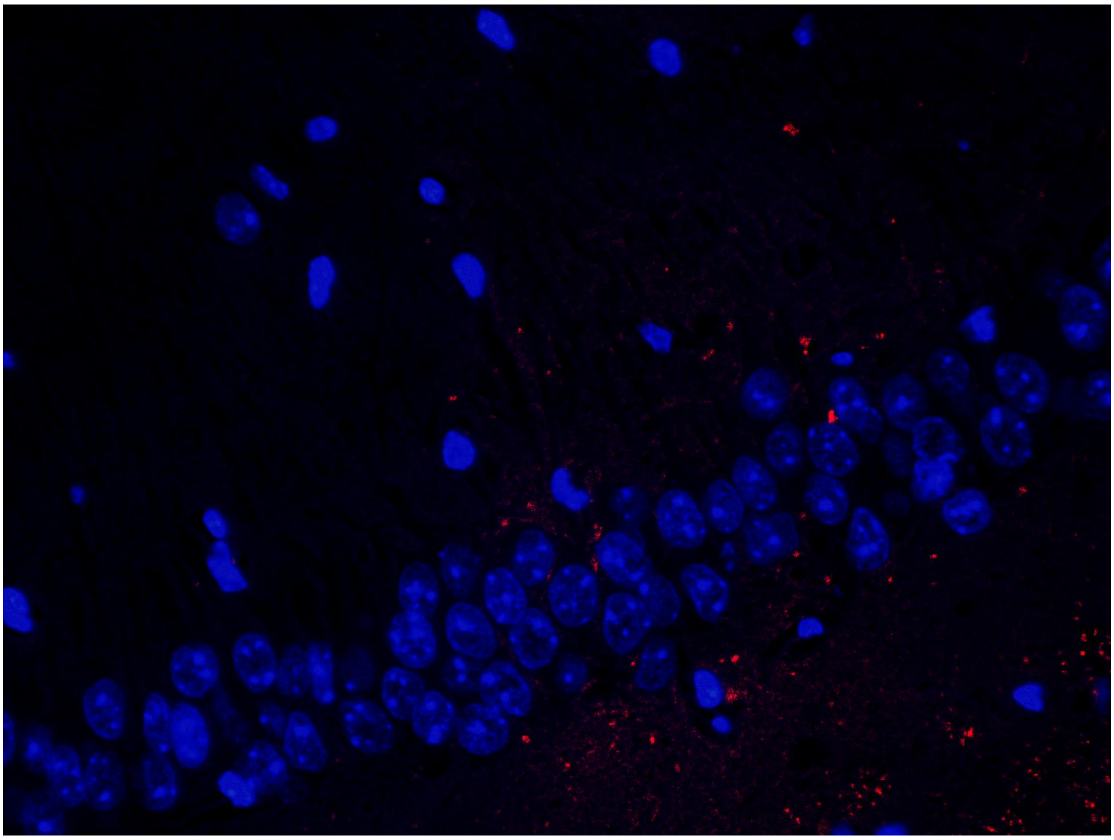
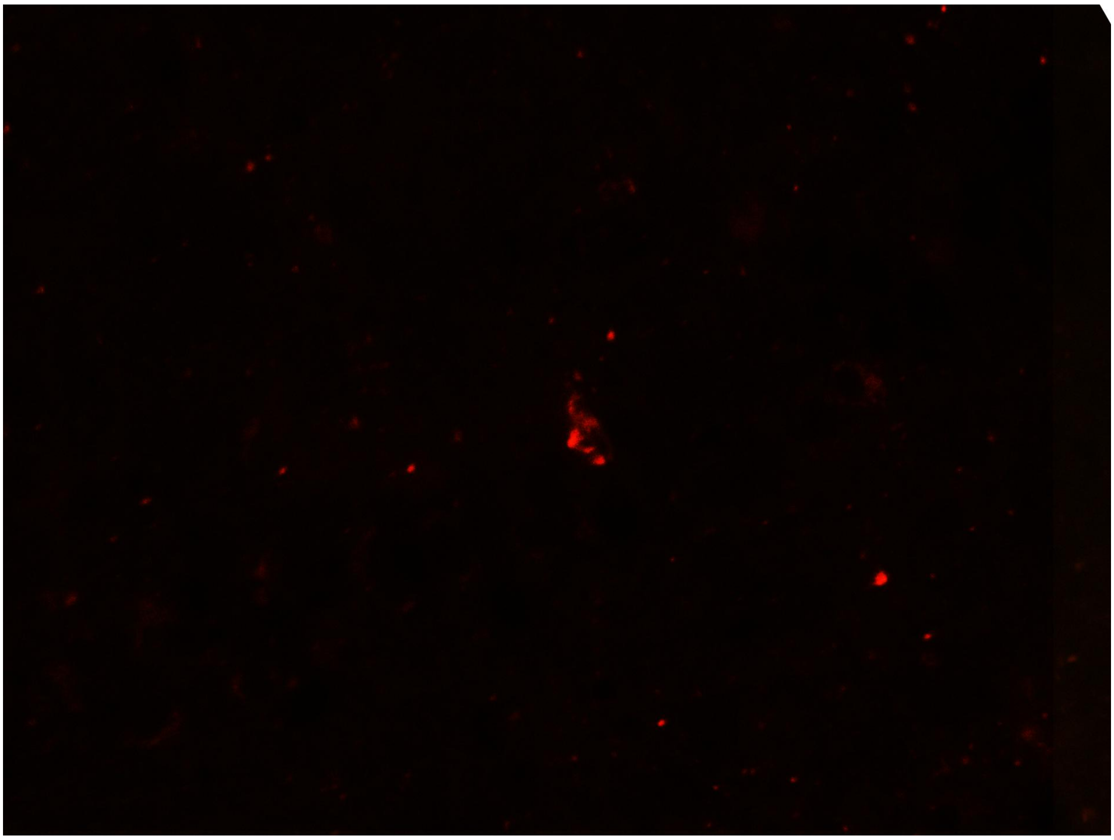

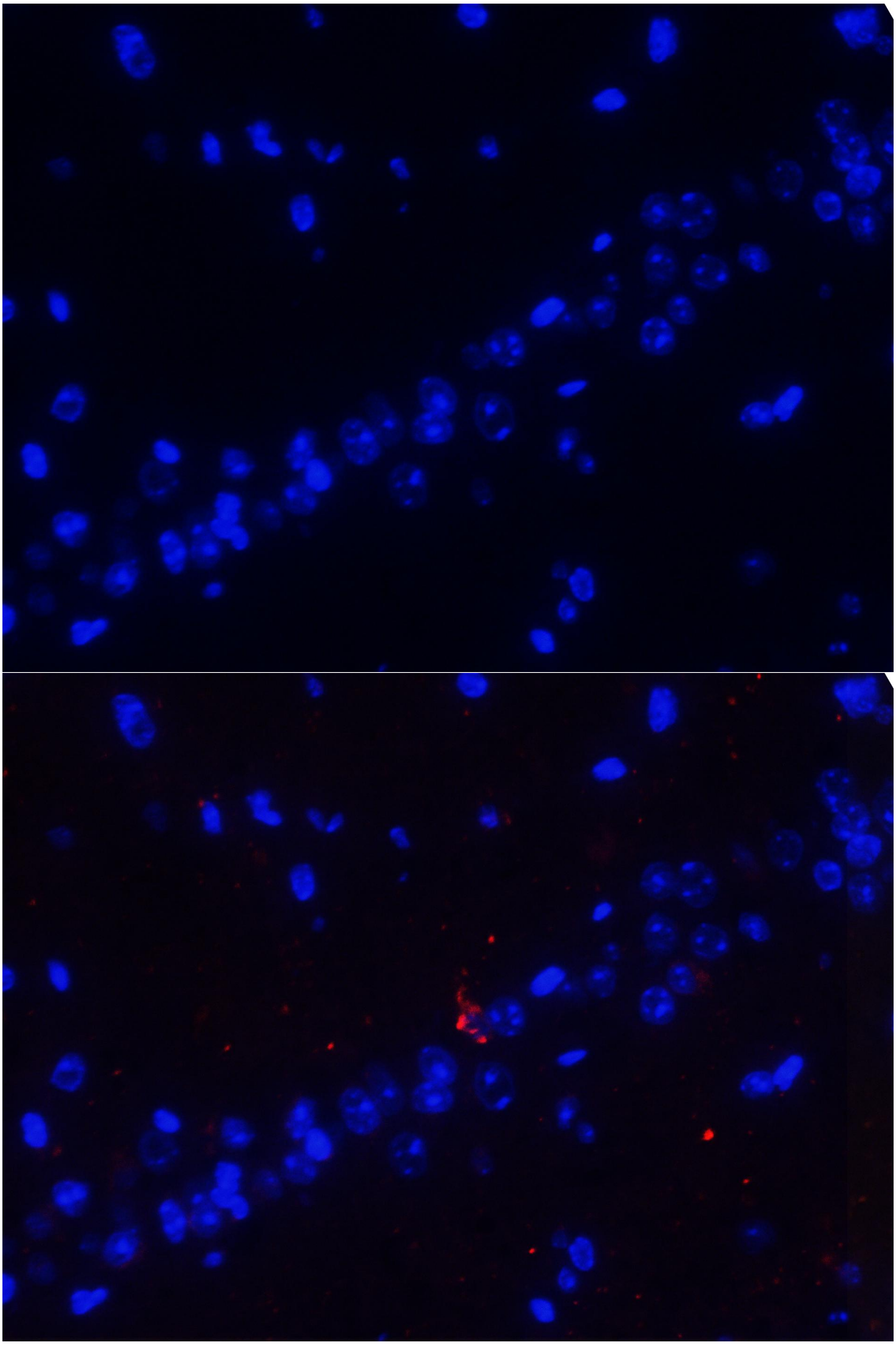

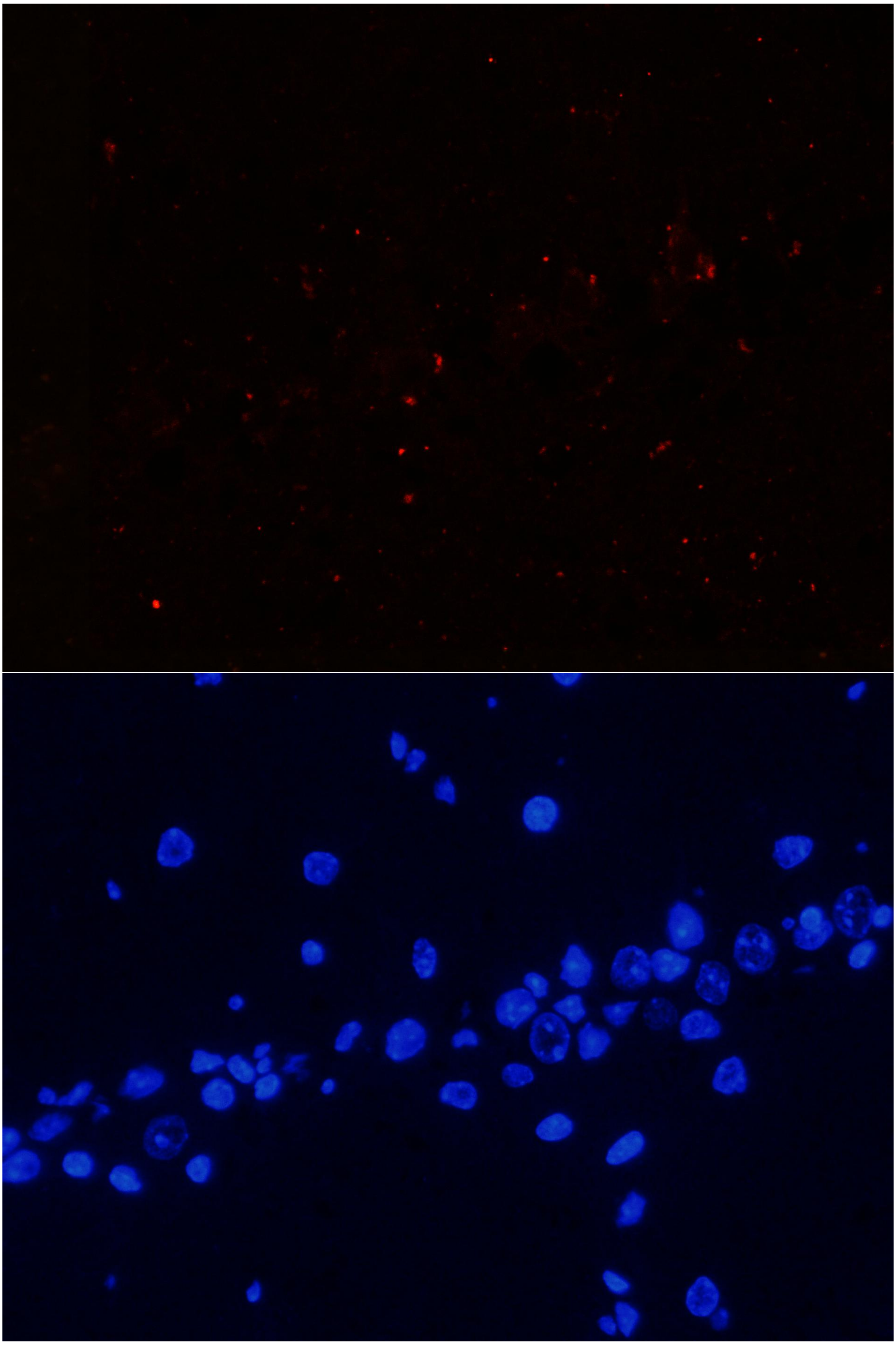

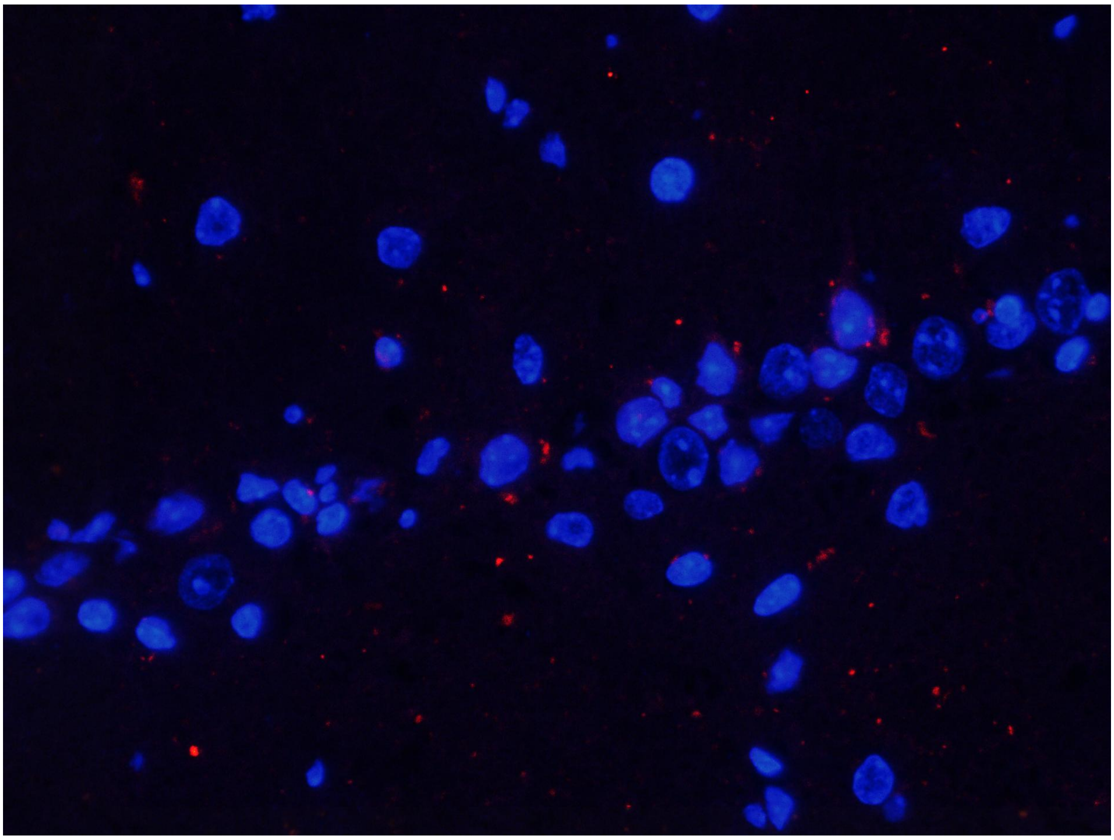

Figure 4, D

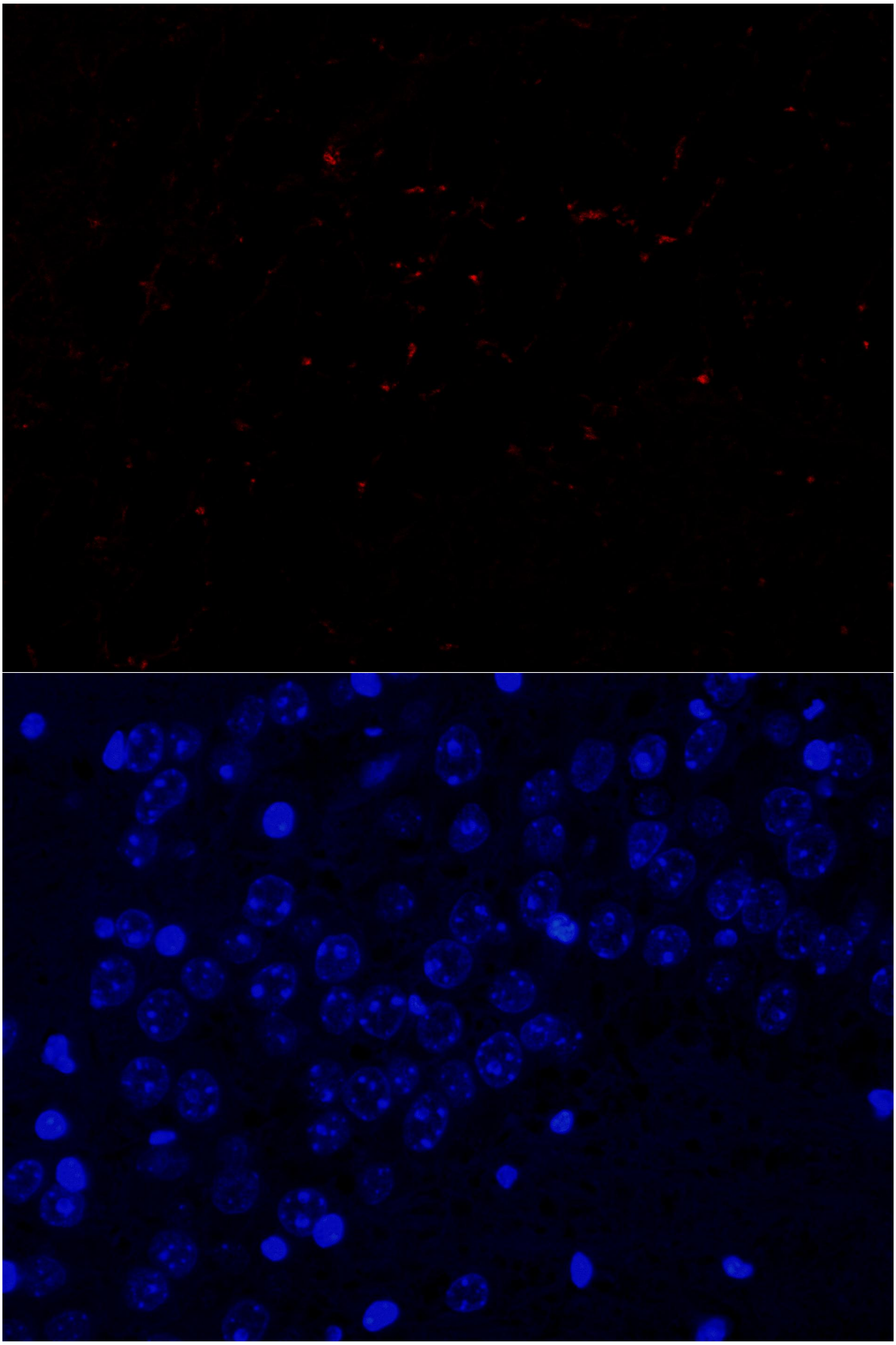

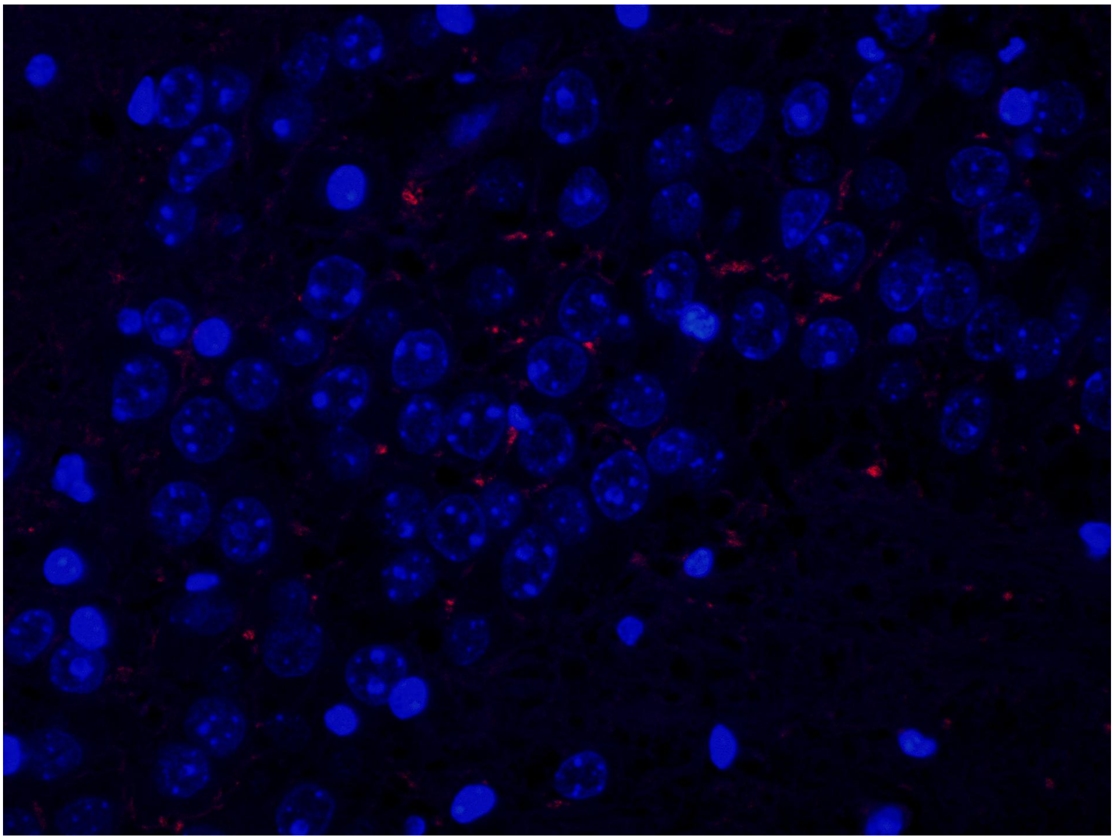

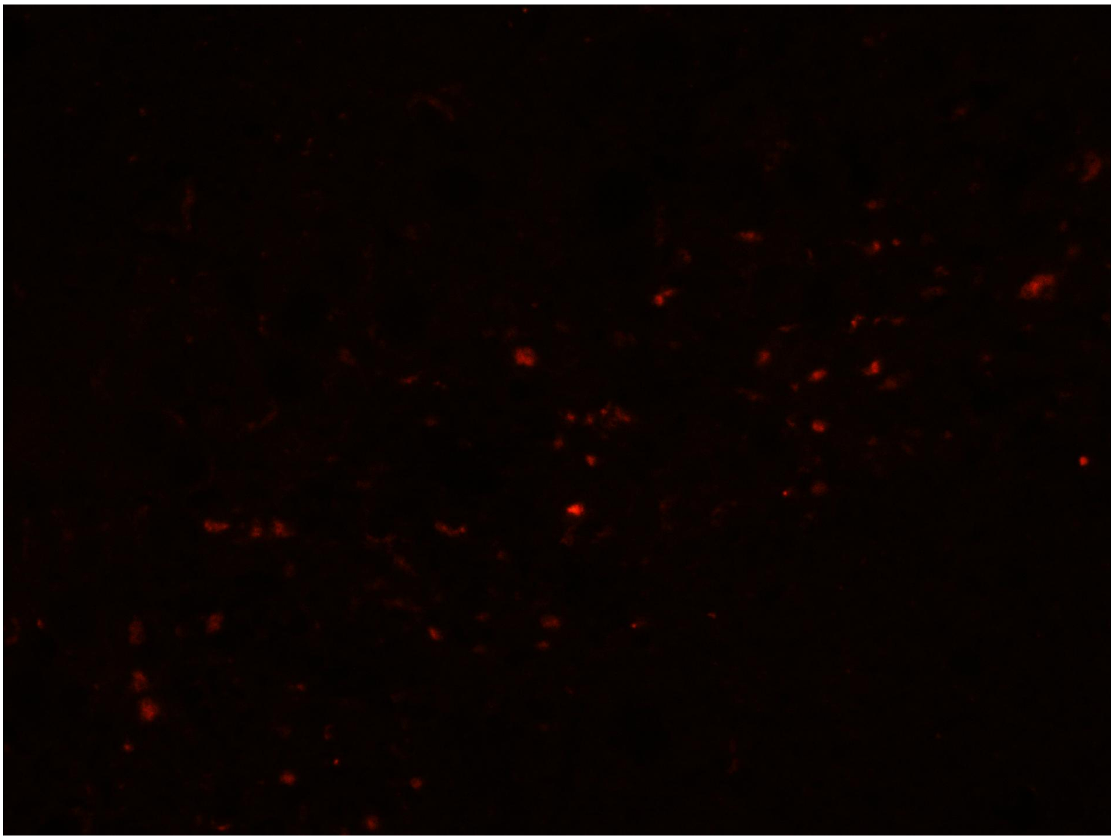

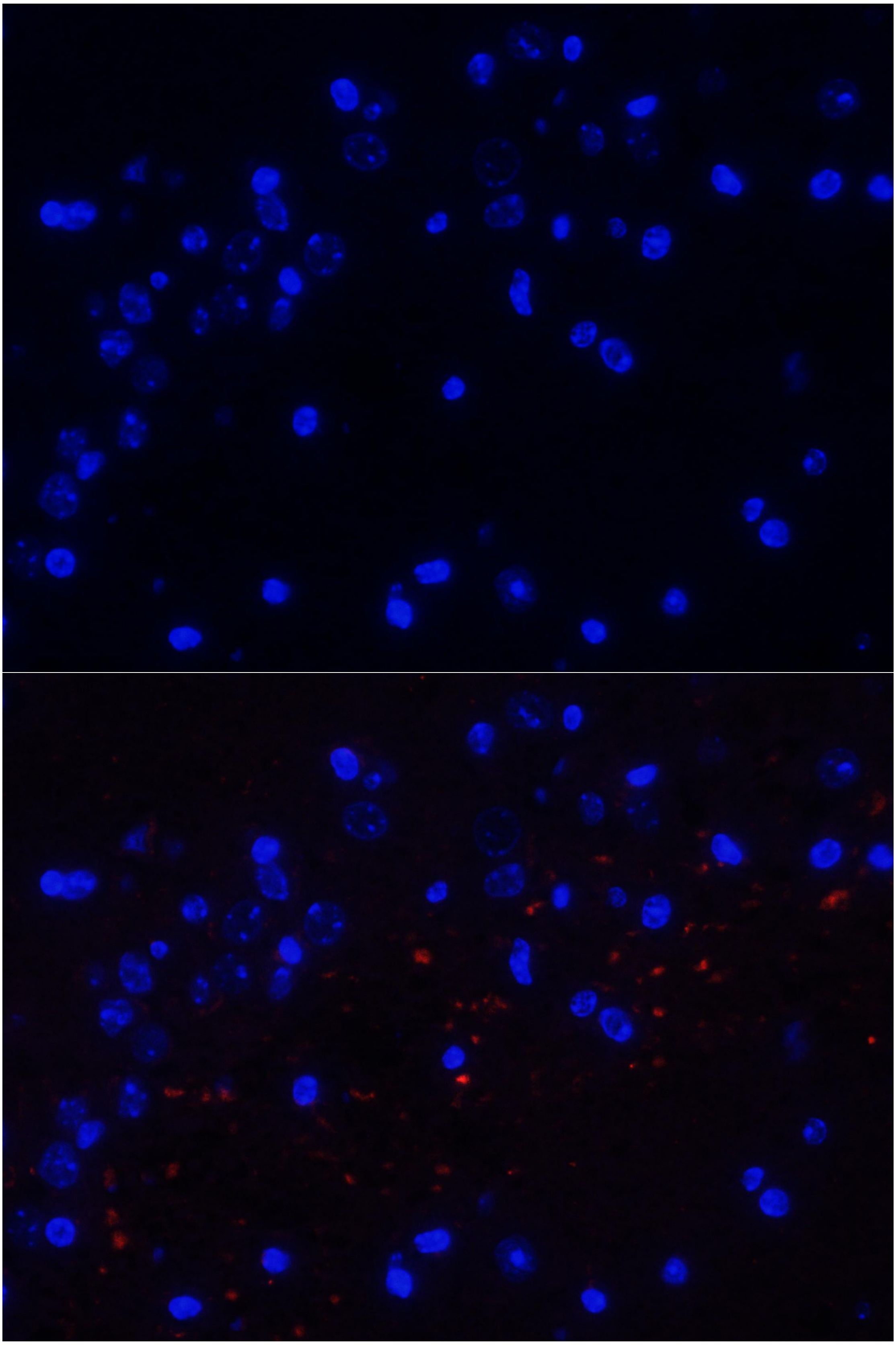

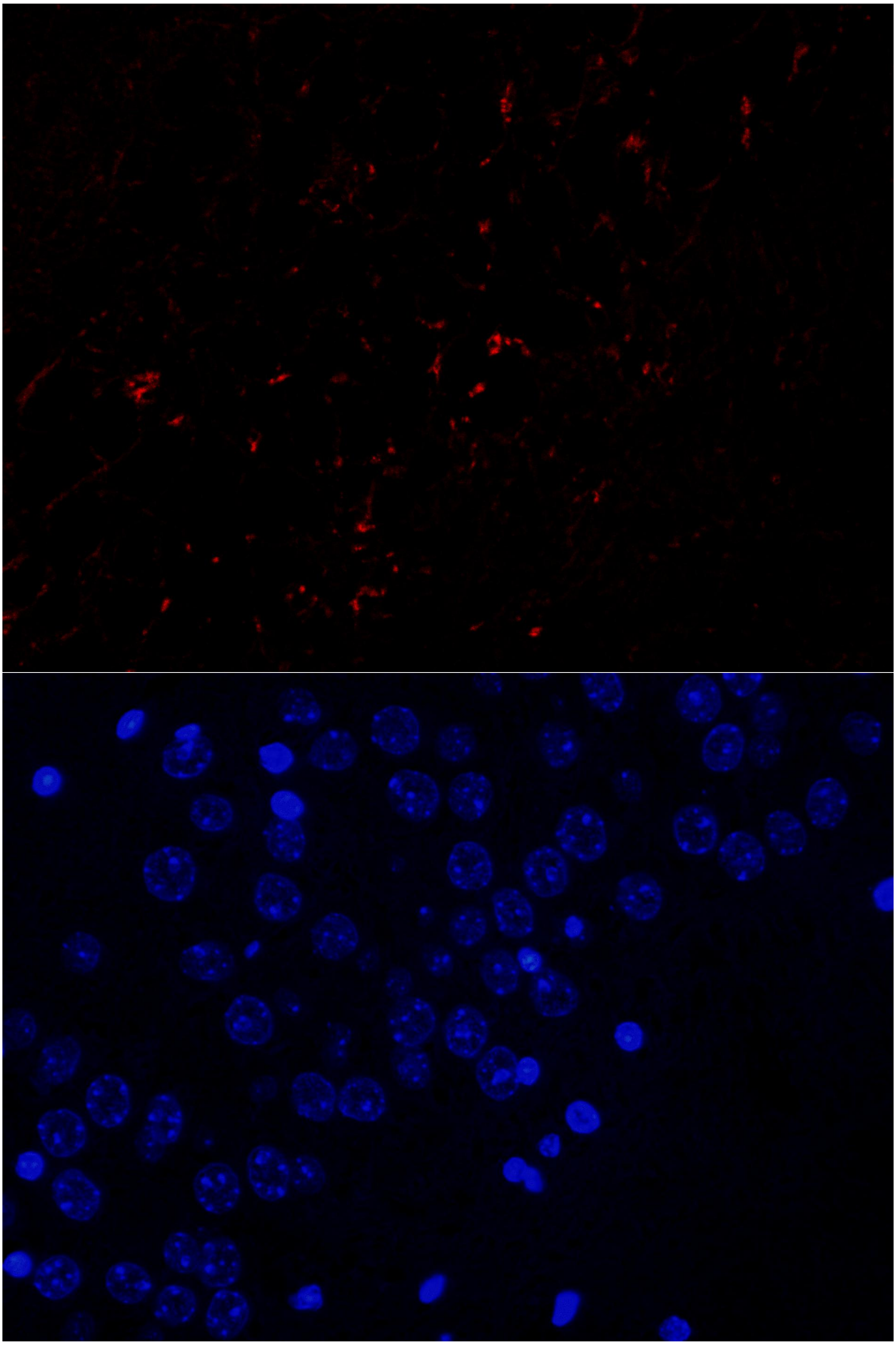

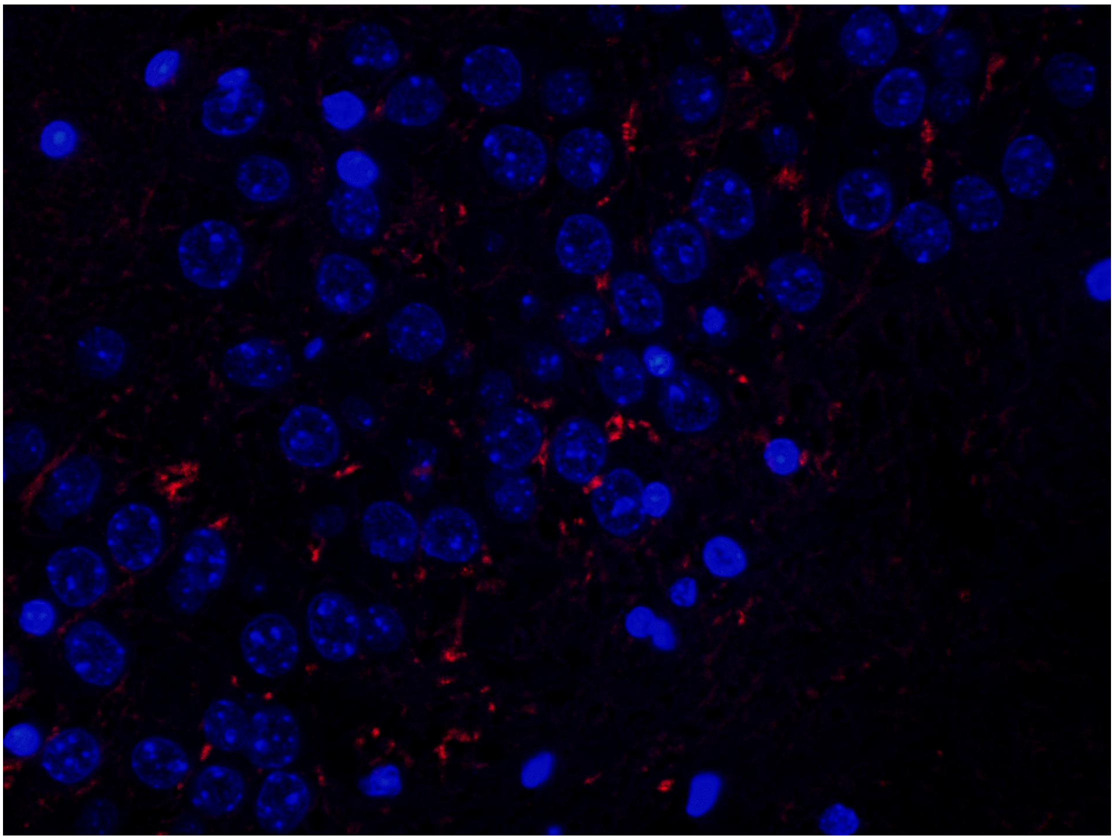

Supplement: Supplementary file 2 [file Data_Sheet_1.PDF]
